# Photonic simulation of entanglement growth and engineering after a spin chain quench

**DOI:** 10.1038/s41467-017-01589-y

**Published:** 2017-11-17

**Authors:** Ioannis Pitsios, Leonardo Banchi, Adil S. Rab, Marco Bentivegna, Debora Caprara, Andrea Crespi, Nicolò Spagnolo, Sougato Bose, Paolo Mataloni, Roberto Osellame, Fabio Sciarrino

**Affiliations:** 1 Istituto di Fotonica e Nanotecnologie—Consiglio Nazionale delle Ricerche (IFN-CNR), P.za Leonardo da Vinci, 32, I-20133 Milano, Italy; 2 Dipartimento di Fisica—Politecnico di Milano, P.za Leonardo da Vinci, 32, I-20133 Milano, Italy; 3 Department of Physics and Astronomy, University College London, Gower Street, WC1E 6BT London, UK; 4 Dipartimento di Fisica—Sapienza, Università di Roma, P.le Aldo Moro 5, I-00185 Roma, Italy

## Abstract

The time evolution of quantum many-body systems is one of the most important processes for benchmarking quantum simulators. The most curious feature of such dynamics is the growth of quantum entanglement to an amount proportional to the system size (volume law) even when interactions are local. This phenomenon has great ramifications for fundamental aspects, while its optimisation clearly has an impact on technology (e.g., for on-chip quantum networking). Here we use an integrated photonic chip with a circuit-based approach to simulate the dynamics of a spin chain and maximise the entanglement generation. The resulting entanglement is certified by constructing a second chip, which measures the entanglement between multiple distant pairs of simulated spins, as well as the block entanglement entropy. This is the first photonic simulation and optimisation of the extensive growth of entanglement in a spin chain, and opens up the use of photonic circuits for optimising quantum devices.

## Abstract (web summary)

The complete maximisation of the entanglement between two complementary blocks of spins due to the dynamics of spin chains remains to be observed. Here, Pitsios et al. simulate such dynamics by propagating single photons in an integrated photonic circuit.

## Introduction

Richard Feynman suggested the idea of a quantum simulator: using a controllable quantum system to mimic the behaviour of a different, more complex one^1^. An important milestone for such a simulator in any platform is to demonstrate the ability to reproduce characteristically quantum features of the model to be simulated. Exactly solvable models of statistical mechanics^2^ are an ideal testbed for benchmarking the simulator, since they provide a plethora of exact results for genuinely quantum attributes, such as the extensive growth of entanglement after a quantum non-equilibrium dynamics. For example, a sudden global change of a many-body Hamiltonian (a quantum quench) can induce a dynamics that ultimately entangles two complementary parts of the system by an amount proportional to their size: a volume law^3–8^ (see ref. ^3^ for an up-to-date review). This quench-induced entanglement growth is not only a phenomenon to benchmark genuinely quantum features of exactly solvable models, but is of pivotal importance in many other areas, as it underpins the ubiquitous process of equilibration^7, 9^, and, through a holographic correspondence, the dynamical formation of black holes in space-time^10, 11^. Entanglement^12^, entanglement growth^13^, and its propagation^14–16^ due to quenches have been verified in atomic/ionic many-body simulators. However, a complete maximisation of the entanglement between two complementary blocks of spins due to the dynamics of spin chains remains to be observed, even in a few-body setting.

In a generic spin chain, after the dynamics following a quench, the entanglement between complementary blocks is proportional to the blocks’ size (volume law). However, it is of highly multipartite form, so that the entanglement between individual spins is vanishing. Even for a Hamiltonian only comprising of permanent nearest-neighbour interactions, the generated entanglement can be tuned, through appropriate engineering of the couplings, into that between individual distant pairs of spins^17, 18^. It is this latter form that is directly useful (without distillation) for, say, quantum teleportation, and thereby for quantum networking. The same high degree of control also enables its usage as ballistic quantum wires^19–23^ and computers^24^. This degree of control in entanglement generation is yet to be achieved in any quantum simulator of condensed matter Hamiltonians. Photonic circuits have been used for realising varied phenomena such as bosonic and fermionic quantum walks^25, 26^, quantum-classical differences in complexity^27–30^, quantum chemistry^31^ and localisation in transport^32^. The ground states of some few-body spin systems^33–35^, as well as some analogues of quantum state transfer (QST) through spin chains using coherent optics^36, 37^, have been realised in photonics.

Here we show, using an appropriately engineered photonic circuit, the capability of photonic devices to simultaneously accomplish the following: (i) the verification of the expected extensive growth of entanglement from the dynamics of a spin chain following a quench, and (ii) the demonstration of a high level of control and optimisation of the generated entanglement, as necessary for building future devices. The simulation of the many-body dynamics is obtained by first mapping the evolution to a continuous time quantum-walk, and then by implementing it via a circuit-based (discrete-step) approach. We demostrate the ability to measure not only the entropy of a block of simulated spins, which would be needed to assess naturally occuring equilibration in generic spin chains that are mappable to free fermions, but also the ability to measure optimised distant entanglement between multiple spin pairs, which would be very difficult in other quantum simulators. Our achievements are made possible by engineering two novel photonic chips as follows: (a) the quantum transport chip and (b) the entanglement characterisation chip. The first chip is designed to implement a spin chain dynamics, which generates multiple entangled pairs of distant “simulated” spins. This pattern is simultaneously of an useful form, as well as one which demonstrates volume law. Entanglement, however, is a notoriously difficult entity to detect even for single pairs and require local measurements in at least two non-commuting bases. Thus, we design the second chip that directly interferes distant output modes of the first chip to make entanglement detection (both the entanglement entropy and the pair-wise spin entanglements) feasible through photon counting. This detection methodology is unique to the field of photonic chips, as it would be extremely difficult to bring several distant atomic paths to interfere.

## Results

### Theory

**Fig. 1:**
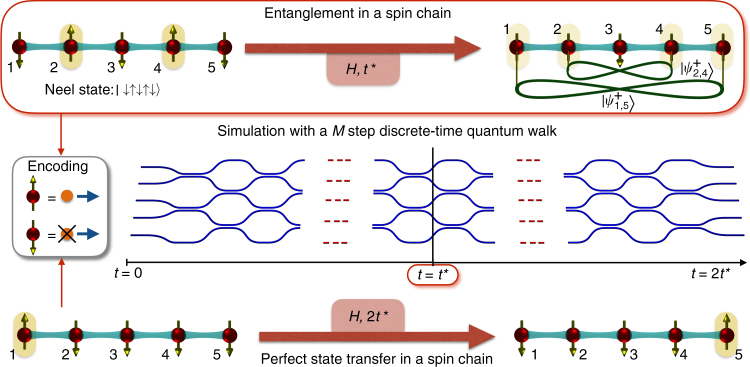
Quantum simulation of spin chain dynamics and the entanglement growth therein in photonic platforms. Fermions simulated on the network map, via the Jordan–Wigner transformation, to spin excitations in a chain: occupied and unoccupied modes correspond respectively to spin up and spin down. The couplings of the effective spin chain Hamiltonian are so engineered as to generate, starting from a Néel state, at a time *t* ^*^, a state with maximal entanglement between distant symmetric sites with respect to the centre of the chain. $\left| {\psi _{i,j}^ + } \right\rangle$ stands for a maximally entangled state of spins in sites *i* and *j*. This rainbow state exhibits a volume law entanglement in which the entanglement between the left and the right halves of the chain is ~*N*/2. The time evolution is approximated through a set of discrete steps (a digital approach). If the dynamics was continued up to time 2*t* ^*^, it would implement an approximately perfect QST

Through our quantum transport chip, we simulate the dynamics induced by a “quantum quench”—a process in which the interactions inside a many-body system are suddenly changed. Let us consider preparing initially the Néel state $\big| {\psi _{{\rm{N}} \acute{\rm e}{\rm e}{\rm l}}} \big\rangle = \big| { \downarrow \uparrow \downarrow ... \uparrow \downarrow } \big\rangle$ of simulated spins, which is one of the ground states of the antiferromagnetic Ising chain and manifestly has no entanglement between any pairs of spins (the whole state is a separable state). In a condensed matter scenario, this state would be prepared by cooling under a Ising Hamiltonian. In photonic technology, we simply inject our apparatus with a simulated Néel state as to be described later. Immediately following the injection, the state starts evolving in accordance to the Hamiltonian simulated by our chip. This corresponds to one of the most extensively theoretically studied spin chain quench^6, 17, 18, 38^ in which the system Hamiltonian is suddenly changed from the Ising model to the XY model

1 $$H = \mathop {\sum}\limits_{i = 1}^{N - 1} \frac{1}{2}{J_i}\left( {\sigma _i^X\sigma _{i + 1}^X + \sigma _i^Y\sigma _{i + 1}^Y} \right),$$

where $\sigma _i^\alpha$ for *α* = *X*, *Y*, *Z* represent the Pauli matrices for the spin in the site *i* and *N* is the length of the chain. Further, the photonics platform enables us to set the ${J_i} = \sqrt {i(N - i)}$ (which has not yet been feasible in the atomic/ionic platforms for quench). Then, one generates, after a relevant time *t* ^*^, a remarkable pattern of nested entangled states^17, 39^, also dubbed as “rainbow states”^40^ (cf Fig. 1):

2 $$\big| {{\psi _{{\rm{v}}{\rm{.l}}{\rm{.}}}}} \big\rangle = \big| {\psi _{1,N}^ + } \big\rangle \big| {\psi _{2,N - 1}^ + } \big\rangle ...,$$

where $\big| {\psi _{i,j}^ + } \big\rangle = \frac{1}{{\sqrt 2 }}( {{{\big| { \uparrow \downarrow } \big\rangle }_{ij}} + {{\big| { \downarrow \uparrow } \big\rangle }_{ij}}} )$. This state corresponds to a “volume law” for entanglement as the number of entangled pairs is ~*N*/2 (exactly *N*/2 for even *N* and (*N* − 1)/2 for the odd *N*). In other words, the amount of entanglement between the left and the right halves of the spin chain generated due to the quench dynamics scales as the size ~*N*/2 of each part. This corresponds to the maximal entropy of entanglement, a much discussed quantity in recent theory and experiment^12^, between the left and the right blocks of the spin chain. Although the “pattern” of the entanglement is not of the multipartite type, as in a generic equilibrated state, in quasi-free models, the dynamical mechanism giving rise to the rainbow state is precisely the same: the propagation of quasiparticles in superposition in opposite directions after the quench^3, 7^. However, note here that the machanism of equilibration in non-integrable systems is quite different^5, 41^. Only a precise interference between the amplitudes of all the quasiparticle propagations, whose mechanism is related to perfect QST^20^, can give rise to the rainbow state. Thus, the “same type of dynamics” as the one behind equilibration can be evidenced by generating and verifying the rainbow state, although the latter has a different form from a generic equilibrated state. Simultaneously, producing the rainbow state demonstrates the ability to optimise the entanglement pattern.

Our simulation technique combines three principal ingredients. Firstly, using the Jordan–Wigner transformation^2, 3, 17, 18^, it is possible to map spin excitations in a chain with nearest-neighbour interactions onto non-interacting fermions hopping in a lattice (hence the name quantum transport chip). Indeed, $H \equiv \mathop {\sum}\nolimits_n {J_n}c_n^\dag {c_{n + 1}} + {\rm{h}}{\rm{.c}}{\rm{.}}$, where the creation/annihilation operators, defined by $c_n^\dag = \sigma _n^ + \mathop {\prod}\nolimits_{j = 1}^{n - 1} ( { - \sigma _j^Z} )$ with $\sigma _n^ + = \left( {\sigma _n^X + i\sigma _n^Y} \right){\rm{/}}2$, satisfy canonical anti-commutation relations. Moreover, as discussed in Supplementary Note 1, in spite of the non-local nature of this transformation, a generic spin state with a fixed number *L* of spin up, $\big| \psi \big\rangle = \mathop {\sum}\nolimits_{{m_1} < \ldots < {m_L}} \psi_{m_1,\dots,m_L} \mathop {\prod}\nolimits_{i = 1}^L \sigma _{{m_i}}^ + \big| { \downarrow \ldots \downarrow } \big\rangle$, can be written as a *L*-fermion state $\big| \psi \big\rangle \equiv \mathop {\sum}\nolimits_{{m_1} < \ldots < {m_L}} \psi_{m_1,\ldots,m_L}\mathop {\prod}\nolimits_{i = 1}^L c_{{m_i}}^\dag \big| 0 \big\rangle$, where we identify a spin up state with the presence of the fermion: $\big| 0 \big\rangle \equiv \big| \downarrow \big\rangle$, $\big| 1 \big\rangle \equiv \big| \uparrow \big\rangle$. In spite of the mapping to a free fermion model, it is customary to regard Eq. (1) as a many-body spin model, in the same sense of quantum Ising model, which has the same feature^2^. Secondly, fermionic behaviour can be simulated in photonic platforms using anti-symmetric configurations of the photons’ internal degrees of freedom; in this respect, two-photon polarisation-entangled states have already been exploited for simulating several phenomena such as Bosonic–Fermionic continuous transition^25, 26^, Anderson localisation^32^ and Fano interference effect in quantum decay^42^. The injection of an anti-symmetric two-photon state in the even modes of the 5-mode interferometer corresponds thus to setting the initial state of the simulator in the equivalent of the 5-sites Néel state. The quenching from the Ising Hamiltonian to the XY Hamiltonian is simulated when the initial state is injected in the quantum transport chip. Third is the fact that, while the spin chain dynamics maps effectively to a continuous time quantum walk of multiple fermions, it can be simulated via discrete quantum walks in the weak transmission limit^43^. Discrete quantum walks provide more accurate control over the implemented Hamiltonian^44^ due to the additional degree of freedom given by the coin operator. An *N*-sites continuous quantum walk for a ‘fixed’ time *t* with site-to-site coupling *J*_*i*_ can be simulated by a photonic network constituted by *M* layers of beamsplitters in cascade (Fig. 1) with transmissivities ${T_i} = {\rm{si}}{{\rm{n}}^{\rm{2}}}\left( {\epsilon {J_i}} \right)$, where $\epsilon = \frac{t}{M}$ is the time step implemented by each layer. The accuracy of this approximation increases with larger number of steps. In essence, this third ingredient is thus a circuit-based approach to quantum simulations as recently undertaken in superconducting networks^45^. Further details on the theoretical model are provided in Supplementary Notes 1–5 and Supplementary Figs. 1–5.

### Experiments

In our work, the quantum transport chip is a 5-sites-, 6-steps-integrated interferometer with engineered couplings for approximating the dynamical generation of the rainbow state of Eq. (2), which displays the volume law. We studied the evolution of a two-particle fermionic state (equivalent to the Néel state of a 5-site antiferromagnet) and its bosonic counterpart, analysing the different statistics at the output and confirming the prediction for creation of the rainbow pattern of entanglement in the fermionic case. This has been possible by merging several experimental techniques such as entanglement generation in bulk optics, state propagation in polarisation-insensitive integrated circuits and entanglement analysis in a 3D tuneable integrated circuit.

### Optical circuits fabrication

**Fig. 2:**
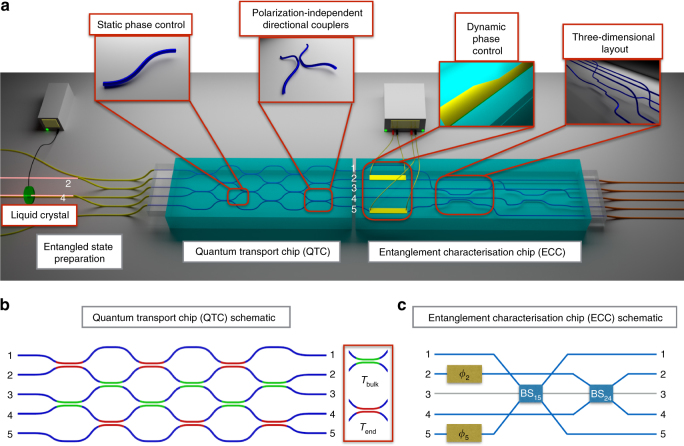
Experimental layout. **a** Representation of the experimental apparatus comprised of the first quantum transport chip and the second entanglement characterisation chip. The insets highlight the specific elements and the three-dimensional geometry of the photonic devices. **b** Schematisation of the quantum transport device of the first chip showing in green the directional couplers of the bulk (with transmissivity *T*_bulk_ = 0.36) and in red those at the edge (with transmissivity *T*_end_ = 0.25) of the device. **c** Schematic representation of the entanglement characterisation device of the second chip, showing the dynamic phase controls *ϕ*_2_ and *ϕ*_5_ acting on the 2nd and 5th waveguides and the 50/50 beam splitters between the (2, 4) and (1, 5) pairs of modes. The 3rd waveguide (in grey) is not involved in any interference processes

The Quantum Transport Chip (QTC) is a 5 input–output waveguide interferometer, encompassing a series of directional couplers as shown in Fig. 2a. In this scheme, each spin particle site is represented by a waveguide, and the directional couplers discretise the interaction between the spins. The spin states at each site are encoded by the presence of a photon for state $\big| \uparrow \big\rangle$ and by the absence of a photon for state $\big| \downarrow \big\rangle$.

In order to properly reproduce the discrete-time quantum state transfer, it is required to use two different transmission values, *T*_bulk_ = 0.36 and *T*_end_ = 0.25, for the internal and the external beam splitters, respectively (Fig. 2b and Supplementary Note 3). This can be achieved by increasing the separation between the two waveguides in the interaction region of the external couplers.

The initial state of the spin chain is prepared by injecting two polarisation-entangled photons (see below). Since the entanglement in the  polarization degree of freedom is exploited to emulate the particle statistics, it should not directly influence the propagation of the two-photon state, thus the chip needs to be polarisation insensitive. This is achieved by means of the tilted geometry design of the directional couplers, allowing equal transmissivity for both horizontal and vertical polarisation^25^.

The entanglement characterisation chip (ECC) is a 5 waveguide device designed with the purpose of evaluating the entanglement generated in the QTC chip (as seen in Fig. 2a). The ECC interferes the signal from the QTC output by using two polarisation insensitive directional couplers between waveguides 1–5 & 2–4. By means of two thermal phase shifters fabricated over waveguides 2 and 5, the chip allows to scan the interference between the two pairs of waveguides by modulating the phase (Fig. 2c)^46, 47^.

The ECC adopts a fully three-dimensional design. The waveguides start at a shallow depth of 25 μm on the collection side, so that the heating elements will operate efficiently. Then they go deeper at 65 μm to form the couplers without any physical crossing of the waveguides (see Fig. 2a inset). The central waveguide is not interacting with any other one.

### Measurements with the quantum transport chip

The simulation of the spin chain dynamics on the photonic platform has been performed by exploiting polarisation-entangled pairs of photons, generated by type-II parametric downconversion process in a beta barium borate (BBO) crystal (see Methods section, Supplementary Note 6 and Supplementary Figs. 6 and 7 for more details). The general expression for the state of the generated photons can be written as $\big| {\Psi _{2{\rm{p}}}^{\,\chi} } \big\rangle { = 2^{ - 1/2}}\left( {\big| {{\rm{HV}}} \big\rangle + {e^{i\chi }}\big| {{\rm{VH}}} \big\rangle } \right)$, where the subscript 2p indicates a two-photon state. The phase parameter *χ* can be controlled by means of an electrically configurable liquid crystal positioned on one of the two-photon paths. Thus both bosonic and fermionic statistics can be simulated by using the Bell states $\big| {\Psi _{2{\rm{p}}}^ + } \big\rangle$ and $\big| {\Psi _{2{\rm{p}}}^ - } \big\rangle$, respectively for their symmetric and anti-symmetric wavefunctions^25^.

The Néel state $\big| { \downarrow \uparrow \downarrow \uparrow \downarrow } \big\rangle$ of the spin chain was simulated by injecting the polarisation-entangled state $\big| {\Psi _{2{\rm{p}}}^ - } \big\rangle$ in the inputs 2 and 4 of the QTC (Fig. 2a, b) through a single mode fibre array and, after evolution into the interferometers, photons are collected via a multimode fiber array and sent to single-photon avalanche photodiodes (APDs). At the output, we collect coincidence measurements from all pairs of outputs to measure the off-diagonal elements (outputs *i* ≠ *j*); for the diagonal contributions (outputs *i* = *j*) an in-fiber beam-splitter is inserted in each single output mode. The same experiment is also performed with $\big| {\Psi _{2{\rm{p}}}^ + } \big\rangle$ to capture the distinctive features of bosonic and fermionic dynamics on the QTC. It is important to observe that this is achievable due to the polarisation insensitivity of the waveguides, and that the input state is insensitive to phase instabilities between two arms of the interferometer. Furthermore, we note that entanglement in two different degrees of freedom is present in the simulator. On one side, polarisation-entanglement is generated by the photon source, and is employed to simulate particles statistics. On the other side, mode entanglement is generated by the QTC as a result of the evolution, and corresponds to the emergence of entanglement between the spins of the simulated chain.

In the limit of a QTC with an infinite number of steps, one would expect a perfect generation of the entangled state of the form (2) exhibiting the volume law at *t* ^*^. A device with 6 steps (whose unitary we denote *U*), when injected with a Néel state, would give unbalanced $\big| {{\psi ^ + }} \big\rangle$ states at symmetric sites (see below). Finally, one has to take into account imperfections of the implemented unitary (Supplementary Note 7), by considering also the possible small residual difference when acting on photons with different polarisations (${\tilde U^{\rm{H}}}$ and ${\tilde U^{\rm{V}}}$).

As a first step, tomographic reconstruction of the unitary transformation matrix by exploiting single-photon and two-photon measurements^48, 49^ has been performed for both polarisations H and V (Supplementary Note 8 and Supplementary Figs. 8–11). The fidelities of the reconstructed unitaries with respect to the expected one, defined as ${{\cal F}^\pi } = | {{\rm{Tr}}\left( {{U^\dag }{{\tilde U}^\pi }} \right)} |{\rm{/}}5$ for *π* = H, V, is found to be ${{\cal F}^{\rm{H}}} = 0.962 \pm 0.001$ and ${{\cal F}^{\rm{V}}} = 0.977 \pm 0.002$, proving the high quality and the polarisation independence of the device.

**Fig. 3:**
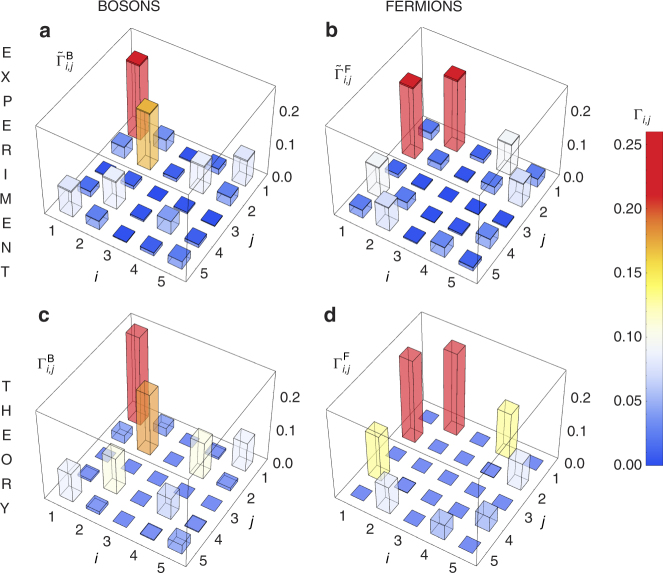
Correlation measurements with the quantum transport chip. **a**, **b** Experimental results of the correlation function, ${\tilde \Gamma _{ij}}$, for bosonic and fermionic transport. Darker parts on top of the bars represent 1*σ* errors for the experimental data, and are due to the poissonian statistics of the coincidence counts. **c**, **d** Theoretical prediction for the correlation function, for bosonic and fermionic transport obtained from the unitary matrix *U* of the transport device. Typical coincidence rates were ~55 per second for all output configurations combined

The results of the quantum dynamics are shown in Fig. 3 for both bosonic and fermionic behaviour, where the experimental $( {\tilde \Gamma _{ij}^{{\rm{B}}/{\rm{F}}}} )$ and theoretical ($\Gamma _{ij}^{{\rm{B}}/{\rm{F}}}$, calculated from the 6-steps unitary *U*) correlation functions^50, 51^ are reported. The output probabilities detected experimentally are related to the correlation function by the equations ${\tilde \Gamma _{ii}} = {P_{ii}}$ and ${\tilde \Gamma _{ij}} = \frac{{{P_{ij}}}}{2}$, due to the symmetrisation of the correlation function matrix. The difference between the two dynamics is clear by observing that bosonic behaviour presents a strong contribution on the diagonal terms, while the latter are suppressed in the fermionic case. Finally, we calculated the similarities between the experimental distributions and the theoretical predictions, defined as ${{\cal S}^{{\rm{B}}/{\rm{F}}}} = {[ {\mathop {\sum}\nolimits_{i,j} {{( {\Gamma _{i,j}^{{\rm{B}}/{\rm{F}}}\tilde \Gamma _{i,j}^{{\rm{B}}/{\rm{F}}}} )}^{1/2}}} ]^2}$, which are found to be ${{\cal S}^{\rm{B}}} = 0.942 \pm 0.005$ and ${{\cal S}^{\rm{F}}} = 0.836 \pm 0.004$.

### Entanglement certification

The time evolution of the state ${\big| {\Psi _{2{\rm{p}}}^ - } \big\rangle _{24}}$ (which simulates the Néel state) through an effective Hamiltonian of the form (1) acting on simulated spins gives a state equal to ${\big| {\psi _{1{\rm{p}}}^ + } \big\rangle _{15}}{\big| {\psi _{1{\rm{p}}}^ + } \big\rangle _{24}}{\big| 0 \big\rangle _3}$ at time *t* ^*^, where ${\big| {\psi _{1{\rm{p}}}^ + } \big\rangle _{ij}}$ denotes the one-photon path-encoded Bell state $\big| {{\psi ^ + }} \big\rangle$ over the modes *i* and *j* ^52^. This state simulates the volume-law-entangled state $\big| {{\psi _{{\rm{v}}{\rm{.l}}{\rm{.}}}}} \big\rangle$ of Eq. (2). As we perform the evolution with a finite number of steps, we get a state of the form $\big| {{\psi _{{\rm{out}}}}} \big\rangle = ( {\alpha {{\big| { \uparrow \downarrow } \big\rangle }_{15}} + \beta {{\big| { \downarrow \uparrow } \big\rangle }_{15}}} )( {\gamma {{\big| { \uparrow \downarrow } \big\rangle }_{24}} + \delta {{\big| { \downarrow \uparrow } \big\rangle }_{24}}} ){\big| \downarrow \big\rangle _3}$. The imbalance between *α* and *β* and between *γ* and *δ*, which can be observed in Fig. 3, comes from the fact that a finite number of discrete step is an approximation of a perfect state transfer. To check the coherence between the terms ${\big| { \uparrow \downarrow } \big\rangle _{ij}}$ and ${\big| { \downarrow \uparrow } \big\rangle _{ij}}$, a phase shift and a beamsplitter transformation over the modes *i* and *j* can be applied, where (*i*, *j*) = (1, 5) and (2, 4). This transformation is implemented in the ECC device shown in Fig. 2c (see Methods section, Supplementary Note 9 and Supplementary Fig. 12 for more details).

**Fig. 4:**
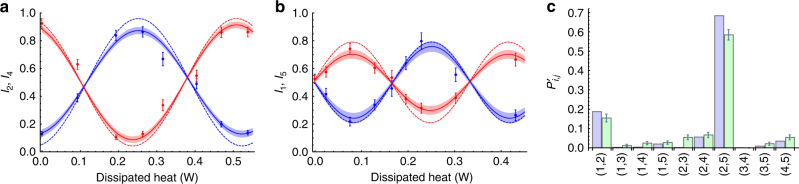
Measurements with the entanglement characterisation chip. **a** Interference fringes for *I*_2_ (blue points) and *I*_4_ (red points) as a function of the dissipated heat on mode 2 (proportional to *ϕ*_2_). **b** Interference fringes for *I*_1_ (blue points) and *I*_5_ (red points) as a function of the dissipated heat on mode 5 (proportional to *ϕ*_5_). In both **a**, **b**, solid lines and shaded areas represent respectively the best fit curves and 1*σ* fit, while the dashed lines corresponds to theoretical predictions. The latter is obtained by considering that the QTC followed by each of the directional coupler of the ECC can be considered as a Mach–Zehnder interferometer. Hence, the adopted best fit model for the experimental data was *a* + *b*cos(*c* + 2π*x*/*d*). **c** Green bars: two-photon probability distribution obtained at the output of the second chip, when the polarisation-entangled anti-symmetric state is injected into input 2 and 4 of the first chip. The distribution is obtained for values of the two phases which maximise $P_{25}^\prime$; blue bars: theoretical predictions. All theoretical models are obtained taking into account the reconstructed matrices of the first chip (${\tilde U^{\rm{H}}}$ and ${\tilde U^{\rm{V}}}$) and the coupling efficiencies at the interface between the two devices (Supplementary Notes 7 and 9). Error bars in the plots are 1*σ* intervals and are due to the poissonian statistics of the coincidence events. Typical coincidence rates in this regime are ~500/h for all output configurations combined

We first measured the two-photon interference fringes obtained by varying the phases *ϕ*_2_ and *ϕ*_5_. More specifically, we defined the quantities *I*_*i*_ (see Methods section), which depend on the effects of one variable phase shift on a single waveguide, and are independent of cross-talk effects by heat dispersion over neighbouring modes. Interference fringes for quantities *I*_1_ and *I*_5_ (*I*_2_ and *I*_4_) obtained when varying phase shift *ϕ*_5_ (*ϕ*_2_) are shown in Fig. 4a, b.

To characterise the degree of entanglement, we first evaluated the entanglement fraction of systems 1–5 and 2–4 with respect to the ideal one-photon Bell state $\big| {\psi _{1{\rm{p}}}^ + } \big\rangle$: ${{\cal E}_{i,j}}{ = _{ij}}\big\langle {\psi _{1{\rm{p}}}^ + } \big|{\rho _{ij}}{\big| {\psi _{1{\rm{p}}}^ + } \big\rangle _{ij}}$, where *ρ*_*ij*_ is the reduced density matrix for two qubits in positions *i* and *j* ^53^. These quantities can be evaluated by measuring the single-photon probabilities *N*′_*i*_ and two-photon probabilities $P_{ij}^\prime$ after evolution through the second device. Indeed, one can find that ${{\cal E}_{1,5}} = N_5^\prime - P_{15}^\prime$ and ${{\cal E}_{2,4}} = N_2^\prime - P_{24}^\prime - P_{23}^\prime + P_{34}^\prime$ (Supplementary Note 4). The first expression implies finding a photon in mode 5 and no photons in mode 1. The second one keeps non-local terms from the mapping, from spin excitations to fermions, which in the case of ${{\cal E}_{1,5}}$ are factorised into a fixed phase term. By evaluating the entanglement fractions from the two-photon probabilities shown in Fig. 4c, we obtain ${{\cal E}_{1,5}} = 0.66 \pm 0.03$ and ${{\cal E}_{2,4}} = 0.74 \pm 0.03.$ This amounts to an approximate verification of Eq. (2) and thereby the growth of entanglement to a volume law.

A further verification of the presence of entanglement, without assuming a specific form for the output state, can be obtained from the measured data. We evaluated the entanglement entropy *S*_A,B_ = *S*(*ρ*_A_) = *S*(*ρ*_B_) of a given bipartition *ρ*_A_/*ρ*_B_ of the chain. The volume law corresponds here to a scaling of *S*_A,B_ ∝ *N*. This quantity can be evaluated directly from the measured data or by exploiting a maximum likelihood approach (see Supplementary Notes 10 and 11 and Supplementary Figs. 13–15 for more details). The second approach permits also to estimate the overall entropy of the state *S*_AB_ = *S*(*ρ*_AB_) and its purity. Here we consider the bipartition A = (1, 2) and B = (3, 4, 5). We obtain *S*_A,B_ = 1.640 ± 0.016 for the entanglement entropy, *S*_AB_ = 0.22 ± 0.05 for the overall entropy and $\tilde \Psi = 0.943 \pm 0.017$ for the purity. From these results it is then possible to evaluate a lower bound on the amount of distillable entanglement by exploiting the hashing inequality^53^, which reads ED_A,B_>*S*_A,B_ − *S*_AB_. The experimental value obtained from the complete set of measured data leads to ${\rm{E}}{{\rm{D}}_{{\rm{A}},{\rm{B}}}} >{\overline {{\rm{ED}}} _{{\rm{A}},{\rm{B}}}} = 1.42 \pm 0.06$.

## Discussion

Using integrated photonics quantum simulation, we show that we can verify the expected growth of entanglement in a spin chain after a quench, as well as its engineering for future applications. The amount of entanglement between the two halves of the spin chain is measured in a 5-site simulated chain after an appropriate time *t* ^*^, and is found to be close to the expected maximal possible value for such a block of spins. The combination of spin chain dynamics, its optimisation to get a rainbow pattern, and the verification of distant entanglement are unique to this photonic realisation. Such results further provide an approach to benchmark the simulator, thus opening up the way for the exploitation of photonic circuits for a significant range of fundamental and practical many-body dynamics investigations. In the future, the technique will allow scaling of such devices to larger spin chains, also enabled by the possibity of fully anti-symmetric states of more photons^26^, for example, using additional degrees of freedom, thus mimicking more fermions. The level of control offered by integrated photonics makes it already feasible to include the capability of dynamically reconfiguring the evolution^46, 47^. Moreover, our methodology of designing the QTC, in particular, a time discretisation of the dynamics that we exploit, makes it amenable to alter the chip design to simulate a large range of such spin chain dynamics—e.g., driven Hamiltonians^54, 55^. Furthermore, by introducing nonlinearities to the waveguides^56^, a larger range of spin Hamiltonians such as those that exhibit thermalisation and are intractable in classical simulations, can be accessed. The principles demonstrated through the ECC, on the other hand, are versatile: both the block entanglement entropy (which can evidence generic many-body entanglement), as well as the entanglement between distant pairs (which depicts its optimisation for usefulness) was measured. Moreover, as we show in Supplementary Note 12, the combination of an ECC chip and photo-detection can be used in the future also in the non-integrable case to measure entanglement by extending the technique developed in refs ^57, 58^. Aside the fundamental interest, the level of control shown by the generation of the rainbow states with a high entangled fraction, will make integrated photonics a suitable platform to benchmark and optimise quantum nano-devices of the future.

## Methods

### Single-photon source

Pairs of entangled single photons were generated by a Spontaneous Parametric Down Conversion (SPDC) process occurring in a 2 mm beta barium borate (BBO) crystal, pumped by a 160 fs pulsed beam at 392.5 nm wavelength. The detected generation rates were ~120 kHz for single-photon counts and ~7 kHz for two-fold coincidences. The phase of the generated entangled state was controlled by means of an electrically-tunable liquid crystal device.

### Devices fabrication technique

In both chips, the QTC and the ECC, the waveguides are inscribed by femtosecond laser writing in Corning Eagle-2000 borosilicate glass. We used a femtosecond Yb:KYW cavity-dumped oscillator at 1030 nm wavelength, emitting pulses with 300 fs duration, and at a 1 MHz repetition rate. The laser beam was focused through a 50×, 0.6 NA microscope objective lens into the glass substrate, which was translated by a computer-controlled three-axis Aerotech FiberGlide 3D series stage, at a velocity of 40 mm/s. In the QTC, the waveguides are inscribed using 220 mW of laser power at a depth of 170 μm. In the ECC, the power of inscription was slightly lower at 210 mW since the waveguides were closer to the surface.

### QTC characterisation

After fabrication, the circuit was characterised classically using a diode laser. The overall performance was verified by collecting the output power distribution for each individual input and comparing it to the theoretically expected one. The polarisation response of the chip was identical for both vertical and horizontal polarisations within 2%, and the similarities between the theoretically expected and experimentally measured output distributions were ${{\cal S}^{\rm{H}}} = 0.963 \pm 0.002$ and ${{\cal S}^{\rm{V}}} = 0.976 \pm 0.002$.

### ECC characterisation

Before using the ECC in the actual quantum experiment, the device was characterised independently by coupling it to a chip containing balanced directional couplers, thus forming Mach–Zehnder interferometers. By applying different voltages to the heating electrodes it was possible to observe the interference fringes at the output of the corresponding interferometers with visibilities *V*_1–5_ = 0.86 ± 0.02 and *V*_2–4_ = 0.94 ± 0.02 (for an expected value of *V*_*i*−*j*_ = 1). In addition, we verified the stability of the thermal tuning of the phase and we have observed that a constant phase value could be maintained for a period of 8 h with a standard deviation ${\sigma _\Phi } = {0.03}$ radians.

### Interference fringes measurement

Let us define ${I_1} = P_{12}^\prime + P_{14}^\prime$, where $P_{ij}^\prime$ is the two-photon probability at the outputs *i* and *j* of the second device. Such quantity depends only from the tuning of phase *ϕ*_5_ on mode 5, while being insensitive to possible thermal cross-talks between the modes. Analogous quantities can be defined for modes 2, 4 and 5 (${I_2} = P_{21}^\prime + P_{25}^\prime$, ${I_4} = P_{41}^\prime + P_{45}^\prime$ and ${I_5} = P_{52}^\prime + P_{54}^\prime$). The recorded visibilities were ${V_{{I_1}}} = 0.51 \pm 0.05$, ${V_{{I_5}}} = 0.40 \pm 0.03$, ${V_{{I_2}}} = 0.74 \pm 0.03$, and ${V_{{I_4}}} = 0.82 \pm 0.03$.

### Data availability

The data sets generated during and/or analysed during the current study are available from the corresponding author on reasonable request.

## Electronic supplementary material

Supplementary Information
