# Supplementary material for: Photonic simulation of entanglement growth and engineering after a spin chain quench
Source: Nat Commun. 2017 Nov 17;8:1569. doi: 10.1038/s41467-017-01589-y (PMC5691163; doi:10.1038/s41467-017-01589-y)
Supplement: Supplementary file 1 — Supplementary Information [file 41467_2017_1589_MOESM1_ESM.pdf]

SUPPLEMENTARY NOTE 1: SIMULATING THE QUENCH DYNAMICS OF SPIN CHAINS

The purpose of this section is to introduce the mapping between spins and fermions, as the latter can be accurately simulated in integrated photonic circuits via a many-particle quantum walk with an anti-symmetric wavefunction.

Spinless fermions and real spin-1/2 systems share some common aspects. Indeed each spin-1/2 is described by a two dimensional space $\{|0\rangle = |\downarrow\rangle, |1\rangle = |\uparrow\rangle\}$. The transition between these two states is obtained via some raising and lowering operators $\sigma^{\pm} = (\sigma^X \pm i\sigma^Y)/2$, being $\sigma^X, \sigma^Y, \sigma^Z$ the Pauli matrices, as $|1\rangle = \sigma^+|0\rangle$ and $|0\rangle = \sigma^-|1\rangle$. In a multi-spin configuration, these raising and lowering operators satisfy fermionic commutation relation on the same site i, namely $\{\sigma_i^+, \sigma_i^-\} = 1$, $(\sigma_i^+)^2 = (\sigma_i^-)^2 = 0$, but, unlike fermionic creation and annihilation operators, these raising and lowering operators commute on different sites, namely $[\sigma_i^\alpha, \sigma_j^\beta] = 0$ for $i \neq j$ and $\alpha, \beta = \pm$. The Jordan-Wigner transformation solves this issue by using a non-local transformation which automatically adds explicit anti-commutation relations on different sites. It reads

$$c_n = \sigma_n^- \prod_{j=1}^{n-1} (-\sigma_j^Z) \ , \qquad\qquad c_n^\dagger = \sigma_n^+ \prod_{j=1}^{n-1} (-\sigma_j^Z) \ . \tag{1}$$

The new operators satisfy the canonical anti-commutation relations, $\{c_n^\dagger, c_m\} = \delta_{nm}$, $\{c_n^\dagger, c_m^\dagger\} = 0$, $\{c_n, c_m\} = 0$, and are therefore truly fermionic creation and annihilation operators.

A generic state of a spin-1/2 system consisting on N spins with a fixed magnetization, namely a fixed number L of spin up, can be written as

$$|\psi\rangle = \sum_{\{m_1, m_2, \cdots, m_L\}} \tilde{\psi}(\{m_n\}) \sigma_{m_1}^+ \sigma_{m_2}^+ \cdots \ \sigma_{m_L}^+ |0\rangle, \tag{2}$$

for some coefficients $\tilde{\psi}(\{m_n\})$ where $|0\rangle$ is a short-hand notation for the many-body vacuum, namely the ferromagnetic state $|0\rangle = |\downarrow\downarrow\cdots\rangle$. Since the raising operators commute on different sites, to avoid multiple counting one can fix the ordering $\{m_1 < \cdots < m_L\}$ and write, without loss of generality, a generic state as

$$|\psi\rangle = \sum_{\{m_1 < \cdots < m_L\}} \psi(\{m_n\}) \sigma_{m_1}^+ \sigma_{m_2}^+ \cdots \ \sigma_{m_L}^+ |0\rangle \ , \tag{3}$$

where the relationship between $\psi(\{m_n\})$ and $\tilde{\psi}(\{m_n\})$ is straightforward. The ordering $\{m_1 < \cdots < m_L\}$ is crucial to write (3) in a simple form as a fermionic state

$$|\psi\rangle = \sum_{\{m_1 < \cdots < m_L\}} \psi(\{m_n\}) c_{m_1}^\dagger c_{m_2}^\dagger \cdots c_{m_L}^\dagger |0\rangle \ . \tag{4}$$

Indeed, starting from $\sigma_{m_L}^+ = c_{m_L}^\dagger \prod_{j=1}^{m_L - 1} (-\sigma_j^Z)$ one sees that the "string" of σ_j^Z operators can be safely removed because $-\sigma_j^Z|0\rangle = |0\rangle$. Similarly, because $m_{L-1} < m_L$, the string of operators in $\sigma_{m_{L-1}}^+$ is composed of σ_j^Z operators with $j \neq m_L$ which commute with $c_{m_L}^\dagger$ as $(-\sigma_j^Z) \equiv e^{i\pi c_j^\dagger c_j}$. By proceeding recursively then one realizes that the two states (3) and (4) describe the same state. Indeed, Eq.(4) is general and holds for any spin state with fixed magnetization. As it will be clear

from the study of the spin dynamics, maintaining the ordering $\{m_1 < \cdots < m_L\}$ in the mapping is crucial to easily interpret the results between the spin and fermionic representation.

A natural model to study the dynamics of a spin chain is the XY model

$$H = \frac{1}{4}\sum_{i=1}^{N-1} J_i \left(\sigma_i^X \sigma_{i+i}^X + \sigma_i^Y \sigma_{i+1}^Y\right) \ . \tag{5}$$

This model has a simple expression in terms of fermionic operators $H = 1/2\sum_i J_i \left(c_i^\dagger c_{i+1} + \text{h.c.}\right)$ and the corresponding Schrödinger evolution in the fermionic picture is described by a Bogoliubov transformation of the creation and annihilation operators: $e^{-itH} c_n^\dagger e^{itH} = \sum_m U_{nm}^t c_m^\dagger$, where $U^t = e^{-it\hat{H}}$ and $\hat{H}$ is the $N \times N$ matrix with elements $\hat{H}_{i,i+1} = \hat{H}_{i+1,i} = J_i/2$. After a certain time t the state of the spin chain is [1]

$$\begin{aligned}|\psi(t)\rangle &= \sum_{\{m_1<\cdots<m_L\}} \sum_{\{\ell_n\}} \psi(\{m_n\}) U_{m_1\ell_1}^t U_{m_2\ell_2}^t \ c_{\ell_1}^\dagger c_{\ell_2}^\dagger \cdots |0\rangle \\ &= \sum_{\{m_1<\cdots<m_L\}} \psi(\{m_n\}) \sum_{\{\ell_1<\cdots<\ell_L\}} \det\{U_{m_j,\ell_k}^t\} \sigma_{\ell_1}^+ \sigma_{\ell_2}^+ \cdots |0\rangle \ ,\end{aligned} \tag{6}$$

where $\det\{U_{m_j,\ell_k}^t\} = \sum_\pi (-1)^\pi \prod_j U_{m_{\pi(j)}\ell_j}^t$ refers to the determinant of a sub-matrix of U and π refers to a permutation of the indices $\{\ell_n\}$. In the above expression we mapped the spin state Eq.(3) to the fermionic representation Eq.(4), used the evolution of the fermionic creation operators to obtain the dynamics, and mapped back the state to the spin representation after an index reordering. This index reordering gives rise to the interesting determinant expression for the time-dependent coefficients.

SUPPLEMENTARY NOTE 2: DISCRETIZATION OF CONTINUOUS TIME QUANTUM WALKS

We consider a configuration as in Supplementary Fig. 1 and we call $|\leftarrow\rangle$ and $|\rightarrow\rangle$ the states moving leftwards or rightwards. The left input of the beam splitter (BS) x is named $|x,\rightarrow\rangle$, while the right input is named $|x,\leftarrow\rangle$. On the other hand, the right output of BS x becomes the left input of BS $x+1$, namely $|x{+}1,\rightarrow\rangle$. Similarly, the left output of BS x is $|x{-}1,\leftarrow\rangle$. In summary,

$$\begin{aligned} |\text{in}, x, \text{Left}\rangle &= |x,\rightarrow\rangle, & |\text{in}, x, \text{Right}\rangle &= |x,\leftarrow\rangle, \\ |\text{out}, x, \text{Left}\rangle &= |x{-}1,\leftarrow\rangle, & |\text{out}, x, \text{Right}\rangle &= |x{+}1,\rightarrow\rangle. \end{aligned} \tag{7}$$

Each beam splitter implements the transformation B, defined as $B|\text{Right}\rangle = t'|\text{Left}\rangle + r'|\text{Right}\rangle, B|\text{Left}\rangle = r|\text{Left}\rangle + t|\text{Right}\rangle$, so in general

$$B_x\,|x,\leftarrow\rangle = t_x'\,|x{-}1,\leftarrow\rangle + r_x'\,|x{+}1,\rightarrow\rangle \ , \quad B_x\,|x,\rightarrow\rangle = r_x\,|x{-}1,\leftarrow\rangle + t_x\,|x{+}1,\rightarrow\rangle \ . \tag{8}$$

We define the evolution operator $U = \oplus_x B_x$ generated by the series of beam splitters. This operator can be split in two terms, $U = SC$, as in the quantum walk literature, where $S = \sum_x |x{-}1\rangle\langle x| \otimes |\leftarrow\rangle\langle\leftarrow| + |x{+}1\rangle\langle x| \otimes |\rightarrow\rangle\langle\rightarrow|$ is the shift operator, and C is a *coin operator* $C|x,\leftarrow\rangle = t_x'|x,\leftarrow\rangle + r_x|x,\rightarrow\rangle, C|x,\rightarrow\rangle = r_x'|x,\leftarrow\rangle + t_x|x,\rightarrow\rangle$, obtained by reshuffling the beam splitter operator. By parametrizing each beam splitter operator with its transmissivity $T_x = t_x^2$

and by defining the angle Θ_x such that $T_x = \sin^2 \Theta_x$, one can show that the coin operator can be written as $C = \exp\left[i\left(\frac{\pi}{2}\mathbb{1} - \hat{\Theta}\right)\sigma^y\right]$, where $\hat{\Theta} = \sum_x \hat{\Theta}_x |x\rangle\langle x|$ and where σ^y is the Pauli matrix acting on the coin space.

To simplify the theoretical analysis we focus on the single-particle subspace and we consider periodic boundary conditions. This boundary condition does not affect the generality of the result for single particles as, for instance, open boundary conditions can be obtained by adding an auxiliary BS with zero transmittance, $T_{\rm Aux} = 0$. Indeed, for the multi-particle sector we will then consider open boundary conditions. Thanks to this, it is then possible to define the Fourier basis $|\tilde{p}\rangle = \frac{1}{\sqrt{N+1}}\sum_x e^{-i\tilde{p}x}|x\rangle$, $\tilde{p} = 2\pi\frac{p}{N+1}$ which diagonalizes the shift operator. Indeed, in terms of the global momentum operator $\hat{P} = \sum \tilde{p}|\tilde{p}\rangle\langle\tilde{p}|$, it is $S = \exp\left(-i\hat{P}\sigma^z\right)$. As interference effects can occur only between even/odd sites, it is convenient to study U^2 and since $e^{i\pi/2\sigma^y}e^{-i\hat{P}\sigma^z}e^{i\pi/2\sigma^y} = -e^{i\hat{P}\sigma^z}$ and $e^{-i\hat{\Theta}\sigma^y} = e^{-i\pi/2\sigma^z}e^{i\hat{\Theta}\sigma^y}e^{i\pi/2\sigma^z}$ one obtains

$$U^2 = -\exp\left(-i\hat{P}\sigma^z\right)\exp\left[-i\hat{\Theta}\sigma^y\right]\exp\left[i\left(\hat{P} - \frac{\pi}{2}\mathbb{1}\right)\sigma^z\right]\exp\left[i\hat{\Theta}\sigma^y\right]\exp\left[i\frac{\pi}{2}\mathbb{1}\sigma^z\right]. \tag{9}$$

The above exact equation is the starting point for the theoretical approximations leading to the simulation of continuous time quantum walks.

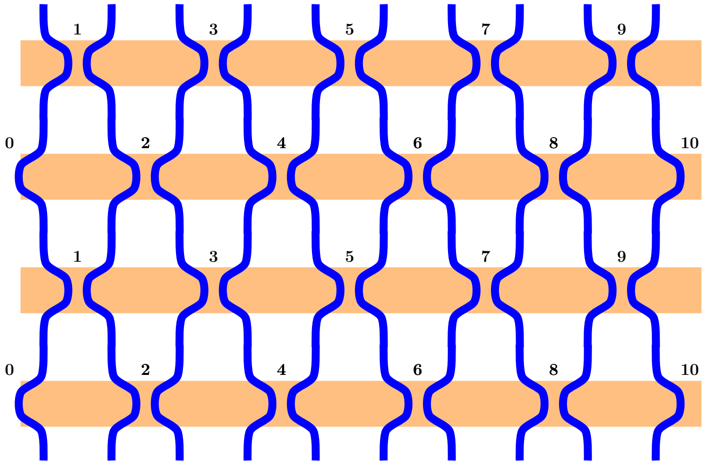

Supplementary Figure 1. Example configuration with 9 sites and 4 time steps. There are also two boundary sites 0,10. The spatial direction corresponds to the horizontal one while the "time" is the vertical direction. Light come from top to bottom. Each beam splitter is localized in a site i.

When $T_x = 1 - \epsilon_x$, with $\epsilon_x \ll 1$, the evolution generated by Eq.(9) in the long-time and long-distance limit can be approximated by the evolution generated by a Dirac Hamiltonian [2,3]. This limit is somehow trivial for the purpose of quantum state transfer. Photons basically move only in one direction, since the reflectivity is negligible, and there is no complex interference phenomena.

The interesting case arises in the opposite limit $T_x \ll 1$, namely when the beam splitters have a low transmittance. In this limit, it has been shown in [2,3] that one can simulate a continuous time quantum walk (CTQW) with a discrete time quantum walk (DTQW). However, those mappings require both $2N$ effective sites for simulating a CTQW with N sites and also require a mixture of

two types of beam splitters, one type with $T_x \approx 1$ and another set with $T_x \approx 0$. Here we use the exact Eq.(9) to derive a simpler mapping. Using a first order expansion for small Θ_x one finds

$$U^2 = -\mathbb{1} + i\, e^{-i\hat{P}\sigma^z} \left[\hat{\Theta}\sigma^y, e^{i\left(\hat{P}-\frac{\pi}{2}\mathbb{1}\right)\sigma^z}\right] e^{i\frac{\pi}{2}\mathbb{1}\sigma^z} + \mathcal{O}(\hat{\Theta}^2) \tag{10}$$

$$= -\mathbb{1} + i \begin{pmatrix} 0 & -i\left(e^{-i\hat{P}}\hat{\Theta}e^{-i\hat{P}} + \hat{\Theta}\right) \\ i\left(e^{i\hat{P}}\hat{\Theta}e^{i\hat{P}} + \hat{\Theta}\right) & 0 \end{pmatrix} + \mathcal{O}(\hat{\Theta}^2)\ , \tag{11}$$

which can be written as

$$U^2 \simeq -W \exp\left(i2\,\hat{H}\,\sigma^z\right) W^\dagger, \qquad W = \frac{1}{\sqrt{2}} \begin{pmatrix} ie^{-i\hat{P}} & -ie^{-i\hat{P}} \\ \mathbb{1} & \mathbb{1} \end{pmatrix}, \tag{12}$$

where we have defined the Hamiltonian as $2H = e^{i\hat{P}}\hat{\Theta} + \hat{\Theta}e^{-i\hat{P}} + \mathcal{O}(\hat{\Theta}^2)$. If $\Theta_x = \epsilon j_x$ where $\epsilon \ll 1$ then

$$H \approx \frac{\epsilon}{2}\sum_x j_x\, |x\rangle\langle x{+}1| + \text{h.c.} \quad j_x \approx \frac{\Theta_x}{\epsilon} \approx \frac{\sqrt{T_x}}{\epsilon}\ . \tag{13}$$

Therefore, the effective evolution reads

$$U(t) \simeq U^t = i^t W\, e^{it\,\hat{H}\,\sigma^z}\, W^\dagger\ . \tag{14}$$

showing that a CTQW can be approximated with a discrete time one.

SUPPLEMENTARY NOTE 3: DISCRETE-TIME QUANTUM TRANSPORT

The dynamics of fully engineered chains [4], where $j_x = \frac{\pi}{N+1}\sqrt{x(N-x)}$, $x = 1, \dots, N-1$ and N is the length of the chain, generates a perfect mirror of the initial state at the transmission time $2t^* = N+1$. Namely, every "walker" initially in position x is perfectly transferred to the position $N-x+1$, after a time $t = 2t^*$. Naively the perfect reconstruction of the wavepacket at the opposite end, without wave dispersion, is due to the mirror symmetry and to the peculiar structure of the spectrum of $\hat{H}$. Indeed, the eigenvalues E_k of this fully engineered Hamiltonian are equispaced, $E_k \propto k$, and due to the resulting constructive interference the initial state is perfectly mapped into its mirror image after the time $2t^*$.

On the other hand, minimally engineered chains [5] require only the engineering of the transmissivity at the boundaries $j_1 = j_{N-1} = j_{\text{opt}}, j_x = 1$ for $x = 2, \dots, N-2$, but allow only a high-quality (almost perfect) transmission between the two ends. These schemes work by maximizing the relevance of the excitations with linear dispersion relation, such that the resulting evolution is almost dispersionless. Indeed, the dispersion relation of the model is $E_k \simeq \cos k$; by flipping the first spin of the chain one creates a distribution of excitations which is centered around $k = \pi/2$, where E_k is almost linear, and has a width that decreases with j_1. Nonetheless, if j_1 is too small the j_1-dependent corrections to the dispersion relation introduce extra non-linearities. The optimal value j_{opt} appears to be the optimal balance between these two effects. For large N there is an approximated formula [5] for the optimal coupling and the transmission time: $j_{\text{opt}} = 1.030\, N^{-1/6}$, while $2t^* = N + 1 + 2.29\, N^{1/3}$.

Simulation of perfect state transfer

We now study the implementation of state transfer in photonic circuits and, for simplicity, we start the discussion with the ideal case, namely perfect state transfer, in the fully engineered case. The purpose of perfect transfer is to transfer a generic state from site 1 to N. In a discrete time-setup we consider also the *coin* degree of freedom and consider an arbitrary superposition of the two possible states on site 1

$$|\text{init}\rangle = \alpha|1,\rightarrow\rangle + \beta|1,\leftarrow\rangle \ , \tag{15}$$

then, as $e^{i\hat{P}}|x\rangle = |x+1\rangle$,

$$W^\dagger|\text{init}\rangle = \frac{\alpha}{\sqrt{2}}(|1,\rightarrow\rangle + |1,\leftarrow\rangle) + i\frac{\beta}{\sqrt{2}}(|2,\rightarrow\rangle - |2,\leftarrow\rangle) \ . \tag{16}$$

Continuous time perfect state transfer can be simulated in M discrete time steps by writing $2t^* = \epsilon M$ for a suitably small ϵ. In other terms, in order to implement a perfect state transfer with M steps one has to chose

$$\epsilon = \frac{2t^*}{M} = \frac{N+1}{M} \ , \tag{17}$$

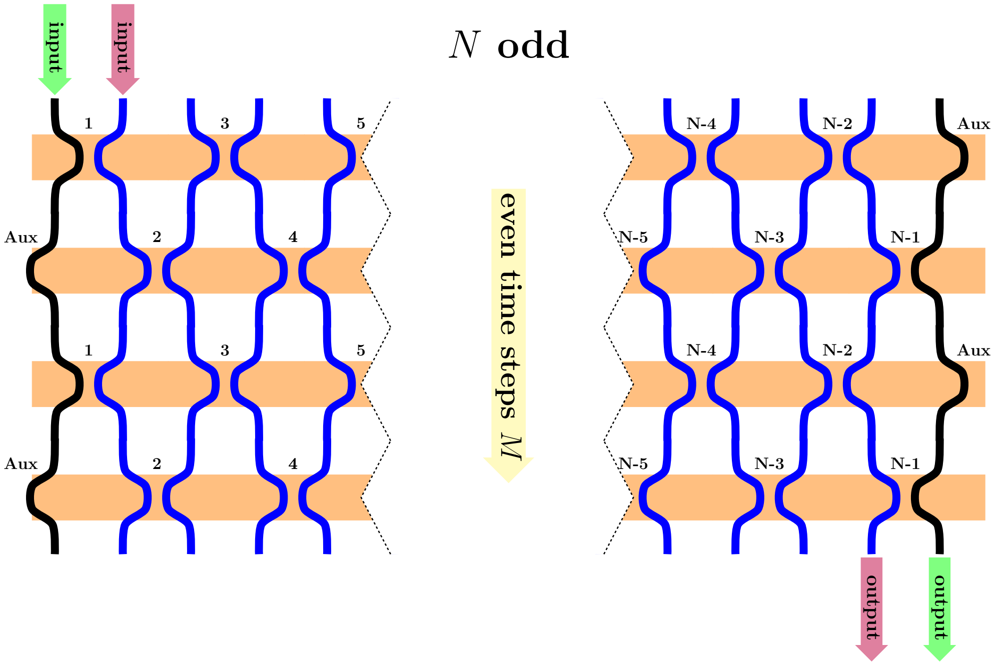

Supplementary Figure 2. Simulation of perfect state transfer with M time steps (M odd).

where $M \gg N$ since ϵ has to be small. The fully engineered Hamiltonian $\hat{H}$ transfers every state in position x to the position $N-x+1$ after the transfer time $2t^*$. Therefore, after M discrete steps

$$e^{iM\hat{H}\sigma^z}\, W|\text{init}\rangle \simeq \frac{\alpha}{\sqrt{2}}(|N,\rightarrow\rangle + |N,\leftarrow\rangle) + i\frac{\beta}{\sqrt{2}}(|N{-}1,\rightarrow\rangle - |N{-}1,\leftarrow\rangle)\ , \tag{18}$$

and

$$|\text{final}\rangle \simeq W\, e^{iM\hat{H}\sigma^z}\, W^\dagger|\text{init}\rangle = \alpha|N,\rightarrow\rangle + \beta|N{-}2,\leftarrow\rangle\ . \tag{19}$$

Note that, because of the definition (7), the states $|N,\rightarrow\rangle$ and $|N{-}2,\leftarrow\rangle$ correspond to the right and left output of the beam splitter in site $N-1$. The derivation presented in this section is a rigorous proof of the conjecture made in [3] where the authors numerically observed that with fully engineered Hamiltonians (i.e. fully engineered coins) even the coin degree of freedom is perfectly transferred.

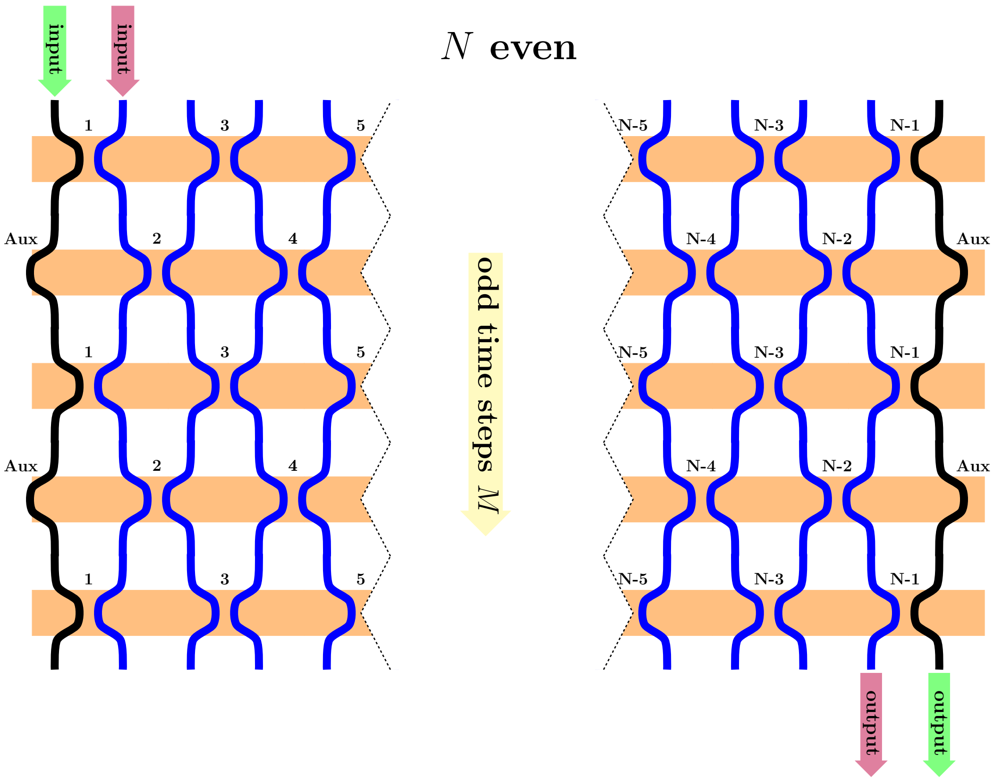

Supplementary Figure 3. Simulation of perfect state transfer with M time steps (M odd).

The overall scheme is presented in the Supplementary Figs. 2 and 3, where the difference between N even and N odd is shown in detail. A single step moves the photon from an even site to an odd site, so an even (odd) number of time steps is required for the transmission when N is odd (even).

As one can see from the Supplementary Figs. 2 and 3, $N-1$ beam splitters are required, per line, for implementing a CTQW (basically a spin chain dynamics) of N sites. Two auxiliary sets

of beam splitters, with $T_{\text{Aux}} = 0$, are required for implementing the the open boundary conditions, though the same result can also be obtained with some mirrors in the auxiliary positions 0 and N.

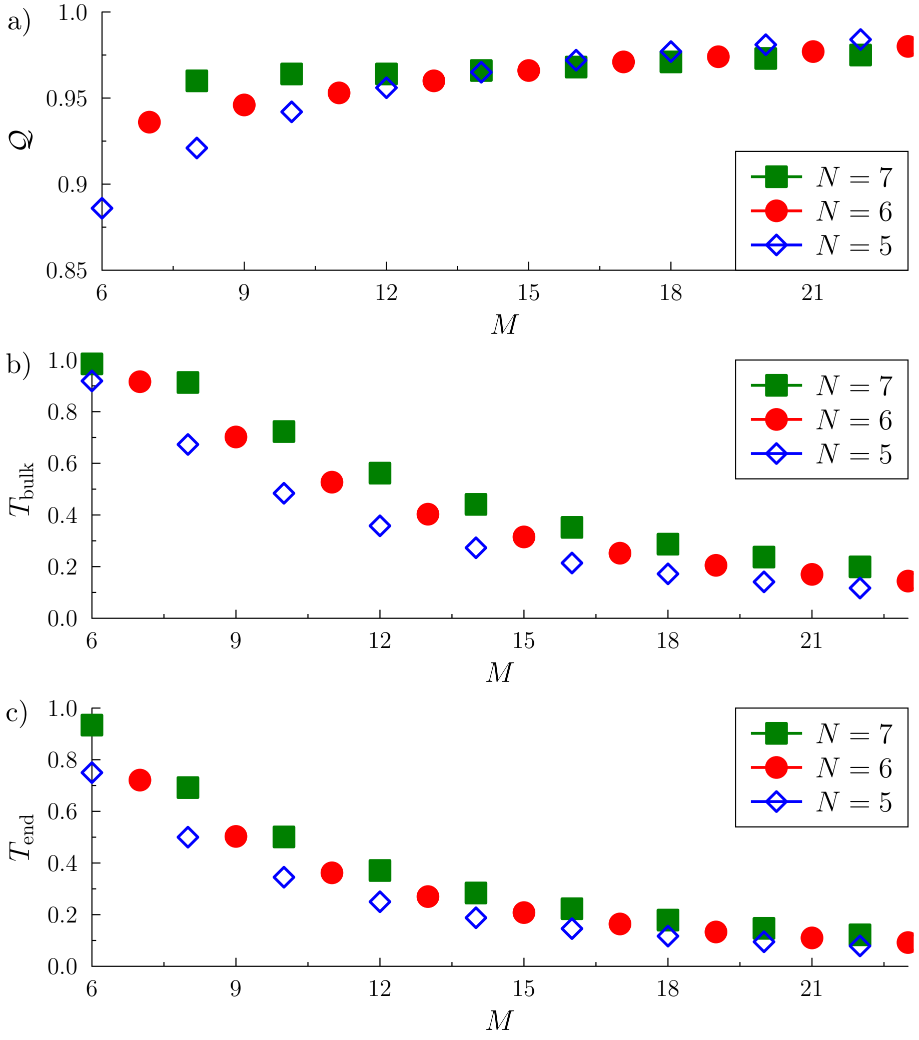

Supplementary Figure 4. Transmission quality (a), transmittance of the beam splitters in the "bulk" (b) T_{bulk} ($T_x = T_{\text{bulk}}$ for $x = 2, \ldots, N-2$), and transmittance of the beam splitters at the ends (c) T_{end} ($T_x = T_{\text{end}}$ for $x = 1, N-1$) for different values of N (horizontal dimension) and M (vertical dimension, namely the number of time steps).

Simulation of state transfer with optimal couplings

When minimally engineered systems are considered, only the "right coin" state $|\rightarrow\rangle$ can be transferred. Indeed, because of the unitary operator W, the left coin state is spread between sites 1 and 2 before the transmission and, unlike fully engineered models, minimally engineered models are not able to reliably transfer a state from site 2 to site $N-1$. For this reason, we consider only the transmission of the right coin (green light in the the Supplementary Figs. 2 and 3). The transmission quality is measured via

$$\mathcal{Q} = \left|\langle N, \rightarrow |U^M|1, \rightarrow\rangle\right|^2, \tag{20}$$

i.e. the probability that a photon goes from $|1,\rightarrow\rangle$ to $|N,\rightarrow\rangle$ after M steps. The results are shown in Supplementary Fig. 4. The quality $\mathcal{Q}$ increases as a function of the number of steps, namely $1-\mathcal{Q}$ decreases polynomially for increasing M. Note that for $N=5$ minimally engineered models and fully engineered ones have the same coupling pattern. In particular, for a fixed transfer time step M, one can evaluate the desired coupling pattern analytically: $T_{\text{end}} = \sin\left(\frac{2\pi}{M}\right)^2$, $T_{\text{bulk}} = \sin\left(\frac{\pi}{M}\sqrt{6}\right)^2$.

SUPPLEMENTARY NOTE 4: ENTANGLEMENT GROWTH AFTER A QUENCH

We consider one of the most common quenches in the spin chain literature, namely we focus on the Hamiltonian

$$\mathcal{H}_\Delta = \sum_n j_n(\sigma_n^x\sigma_{n+1}^x + \sigma_n^y\sigma_{n+1}^y + \Delta\sigma_n^z\sigma_{n+1}^z) , \tag{21}$$

and we consider the quench from $\Delta=\infty$ to $\Delta=0$. We assume that initially the system is in (one of) the ground states of $\mathcal{H}_\infty$, namely the Néel state $|\downarrow\uparrow\downarrow\uparrow\downarrow\uparrow\downarrow\ldots\rangle$, and then we effectively switch off the parameter Δ so that the system starts to evolve according to the XY Hamiltonian $\mathcal{H}_0$. One can show [6] that when the couplings j_n are engineered for perfect state transfer, then after half the transfer time, namely after t^*, the initial state $|\downarrow\uparrow\downarrow\uparrow\downarrow\uparrow\downarrow\ldots\rangle$ evolves into the state $|\psi^+_{1,N}\rangle|\psi^+_{2,N-1}\rangle|\psi^+_{3,N-2}\rangle\cdots$, where $|\psi^+\rangle \propto |\uparrow\downarrow\rangle + |\downarrow\uparrow\rangle$ and the subscripts index the spins of the chain. Hence, after half the transmission time, two opposite spins lying at the same distance from the boundaries become maximally entangled. This corresponds to the creation of $N/2$ Bell pairs, namely the maximal amount of pair-wise entanglement.

In view of the Jordan-Wigner transformation, the many-spin anti-ferromagnetic initial state can be simulated using $N/2$ photons in an antisymmetric configuration. We focus on the case where N is odd, though the same analysis can be performed when N is even. The rainbow state can be written in the fermionic form using the relationship between Eq.(3) and Eq.(4). Indeed, if $N=2L+1$,

$$|\psi_{\text{rainbow}}\rangle = |\psi^+_{1,N}\rangle|\psi^+_{2,N-1}\rangle|\psi^+_{3,N-2}\rangle\cdots , \tag{22}$$

$$= \left(\frac{\sigma_1^+ + \sigma_N^+}{\sqrt{2}}\right)\left(\frac{\sigma_2^+ + \sigma_{N-1}^+}{\sqrt{2}}\right)\left(\frac{\sigma_3^+ + \sigma_{N-2}^+}{\sqrt{2}}\right)\cdots|0\rangle \tag{23}$$

$$= \left(\frac{c_1^\dagger - (-1)^L c_N^\dagger}{\sqrt{2}}\right)\left(\frac{c_2^\dagger + (-1)^L c_{N-1}^\dagger}{\sqrt{2}}\right)\left(\frac{c_3^\dagger - (-1)^L c_{N-2}^\dagger}{\sqrt{2}}\right)\cdots|0\rangle , \tag{24}$$

the different signs appear when the ordering of (4) is removed to write the state in the convenient form (24). We now call BS_{ab} the transformation that does a beam splitter operation on modes a

and b, namely

$$c_a^\dagger \to \frac{c_a^\dagger + c_b^\dagger}{\sqrt{2}} \ , \tag{25}$$

$$c_b^\dagger \to \frac{c_a^\dagger - c_b^\dagger}{\sqrt{2}} \ . \tag{26}$$

It is now simple to show that

$$|\psi_{\rm rainbow}\rangle = \begin{cases} \mathrm{BS}_{1,N}\mathrm{BS}_{2,N-1}\cdots\mathrm{BS}_{L,L+2}c_2^\dagger c_4^\dagger c_L^\dagger c_{L+3}^\dagger c_{L+5}^\dagger \cdots c_N^\dagger|0\rangle & \text{if } L \text{ is even,} \\ \mathrm{BS}_{1,N}\mathrm{BS}_{2,N-1}\cdots\mathrm{BS}_{L,L+2}c_1^\dagger c_3^\dagger c_L^\dagger c_{L+3}^\dagger c_{L+5}^\dagger \cdots c_{N-1}^\dagger|0\rangle & \text{if } L \text{ is odd,} \end{cases} \tag{27}$$

namely that the rainbow state can be generated from a product state via the application of nested 50/50 beam splitters $\mathcal{B} = \mathrm{BS}_{1,N}\mathrm{BS}_{2,N-1}\cdots\mathrm{BS}_{L,L+2}$. From the previous expression it is now clear that the fidelity between the rainbow state and a generic state $|\psi\rangle$ can be calculated from (4) as

$$\mathcal{F} = |\langle\psi_{\rm rainbow}||\psi\rangle\rangle|^2 = \begin{cases} |\psi'(2,4,\ldots,L,L+3,L+5,\ldots,N)|^2 & \text{if } L \text{ is even,} \\ |\psi'(1,3,\ldots,L,L+3,L+5,\ldots,N-1)|^2 & \text{if } L \text{ is odd,} \end{cases} \tag{28}$$

where $|\psi'\rangle = \mathcal{B}^\dagger|\psi\rangle$ and $\psi'(\{m_n\})$ refers to the coefficients of $|\psi'\rangle$ in the decomposition (4). Therefore, to measure the fidelity between a generic state and the rainbow state one has to apply a series of beam splitters between independent pairs of modes and then measure the joint probability to have the L particles in certain specific modes, namely modes $(2,4,\ldots,L,L+3,L+5,\ldots,N)$ for L even or modes $(1,3,\ldots,L,L+3,L+5,\ldots,N-1)$ for L odd.

While the *global* fidelity between the output state and the rainbow state has a quite simple form, the fidelity between each pair of spins $(x, N-x+1)$, which are maximally entangled in $|\psi_{\rm rainbow}\rangle$, and the Bell state $|\psi^+_{x,N-x+1}\rangle$ may be quite complicated because of the non-local nature of the Jordan-Wigner transformation. This quantity is called entanglement fraction and is defined as $\mathcal{E}_{i,j} = \langle\psi^+|\rho_{ij}|\psi^+\rangle$, where ρ_{ij} is the density matrix obtained by tracing out all sites but i and j. We will show later how to measure the different entanglement fractions in the specific 5-mode configuration of our experimental setup. Here on the other hand we show that there is a simple expression to measure $\mathcal{E}_{1,N}$ between the distant pair $(1,N)$, which is an important quantity to detect long-distant entanglement. By using the definition we may write

$$\mathcal{E}_{1,N} = \langle\psi^+_{1,N}|\mathop{\rm Tr}_{2,3,\ldots,N-1}[|\psi\rangle\langle\psi|]|\psi^+_{1,N}\rangle = \langle\psi|\left[|\psi^+_{1,N}\rangle\langle\psi^+_{1,N}|\otimes \mathbb{1}_{2,3,\ldots,N-1}\right]|\psi\rangle \ . \tag{29}$$

While similar expressions hold also for generic pairs of spins, a simple explicit form for the pair $(1,N)$ is obtained by exploiting the identity $c_N^\dagger = \sigma_N^+\mathcal{P} = -\mathcal{P}\sigma_N^+$ where $\mathcal{P} = \prod_{j=1}^N(-\sigma_j^Z)$ is parity of the whole chain, which is a conserved quantity. In particular, starting from the Néel state the parity is constant during the dynamics and it is equal to $(-1)^L$. Thus we can write

$$\mathcal{E}_{1,N} = \frac{1}{2}\langle\psi|\left[(\sigma_1^+ + \sigma_N^+)|0_1 0_N\rangle\langle 0_1 0_N|(\sigma_1^- + \sigma_N^-)\otimes\mathbb{1}_{2,3,\ldots,N-1}\right]|\psi\rangle \ . \tag{30}$$

$$= \frac{1}{2}\langle\psi|\left[(c_1^\dagger - \mathcal{P}c_N^\dagger)|0_1 0_N\rangle\langle 0_1 0_N|(c_1 - c_N\mathcal{P})\otimes\mathbb{1}_{2,3,\ldots,N-1}\right]|\psi\rangle \ . \tag{31}$$

$$= \frac{1}{2}\langle\psi|\left[(c_1^\dagger - (-1)^L c_N^\dagger)|0_1 0_N\rangle\langle 0_1 0_N|(c_1 - (-1)^L c_N)\otimes\mathbb{1}_{2,3,\ldots,N-1}\right]|\psi\rangle \ . \tag{32}$$

If L is odd then $(c_1^\dagger - (-1)^L c_N^\dagger) = \mathrm{BS}_{1,N} c_1^\dagger$ while for L even $(c_1^\dagger - (-1)^L c_N^\dagger) = \mathrm{BS}_{1,N} c_N^\dagger$. We focus on L even since the other case can be obtained with a similar method. As before we call $|\psi'\rangle = \mathcal{B}^\dagger|\psi\rangle$, then $\mathcal{E}_{1,N} = \langle\psi'|c_N^\dagger|0_1 0_N\rangle\langle 0_1 0_N|c_N|\psi'\rangle$. The final expression is then obtained using $|0_1 0_N\rangle\langle 0_1 0_N| = c_1 c_1^\dagger c_N c_N^\dagger$ and therefore

$$\mathcal{E}_{1,N} = \begin{cases} \langle\psi'|c_N^\dagger c_N c_1 c_1^\dagger|\psi'\rangle & \text{if } L \text{ is even,} \\ \langle\psi'|c_1^\dagger c_1 c_N c_N^\dagger|\psi'\rangle & \text{if } L \text{ is odd.} \end{cases} \tag{33}$$

The entanglement fraction between other pairs of spins can be obtained with a similar method, for instance

$$\mathcal{E}_{L,L+2} = \frac{1}{2}\langle\psi|\left[(\sigma_L^+ + \sigma_{L+2}^+)|0_L 0_{L+2}\rangle\langle 0_L 0_{L+2}|(\sigma_L^- + \sigma_{L+2}^-)\right]|\psi\rangle \tag{34}$$

$$= \frac{1}{2}\langle\psi|\left[(c_L^\dagger + \sigma_L^Z \sigma_{L+1}^Z c_{L+2}^\dagger)|0_L 0_{L+2}\rangle\langle 0_L 0_{L+2}|(c_L + \sigma_L^Z \sigma_{L+1}^Z c_{L+2})\right]|\psi\rangle. \tag{35}$$

If we apply the transformation $\mathcal{B}$ and set $D^\dagger = (c_L^\dagger c_{L+1} c_{L+1}^\dagger - c_{L+1}^\dagger c_{L+1} c_{L+2}^\dagger)$ then

$$\mathcal{E}_{L,L+2} = \langle\psi'|\left[D^\dagger|0_L 0_{L+2}\rangle\langle 0_L 0_{L+2}|D\right]|\psi'\rangle \tag{36}$$

$$= \langle\psi'|\left[D^\dagger c_L c_L^\dagger c_{L+2} c_{L+2}^\dagger D\right]|\psi'\rangle \tag{37}$$

$$= \langle\psi'|\left[c_L^\dagger c_L c_{L+1} c_{L+1}^\dagger c_{L+2} c_{L+2}^\dagger + c_L c_L^\dagger c_{L+1}^\dagger c_{L+1} c_{L+2}^\dagger c_{L+2}\right]|\psi'\rangle\ . \tag{38}$$

Setting then $n_j = c_j^\dagger c_j$ we find the simple final expression

$$\mathcal{E}_{L,L+2} = \langle\psi'|\left[n_L - n_L n_{L+1} - n_L n_{L+2} + n_{L+1} n_{L+2}\right]|\psi'\rangle\ , \tag{39}$$

which can be readily obtained via measuring correlation functions as shown in the next section. As it is clear from the final form, because of the non-local nature of the Jordan-Wigner transformation, $\mathcal{E}_{L,L+2}$ depends also on the state of the $(L+1)$-th mode.

Experimental setting

We focus on a chip with $N = 5$ modes and, as a consequence, the number of particles to produce a rainbow state is $L = 2$. Writing the time evolution explicitly for bosons and fermions one can show that after half the transmission time

$$|\psi(0)\rangle = c_2^\dagger c_4^\dagger|0\rangle \quad \longrightarrow |\psi(t^*)\rangle \propto \begin{cases} (c_1^\dagger - c_5^\dagger)(c_2^\dagger + c_4^\dagger)|0\rangle & \text{for Fermions,} \\ \\ \left[(c_1^\dagger - c_5^\dagger)^2 + (c_2^\dagger + c_4^\dagger)^2\right]|0\rangle & \text{for Bosons,} \end{cases} \tag{40}$$

where we used $c_j^\dagger$ to denote either a bosonic or fermionic creation operator. Therefore, the resulting interference pattern is completely different: while for bosons the modes are correlated pairwise (namely mode 1 with mode 5, and mode 2 with mode 4), in the fermionic case the resulting state corresponds to a delocalized particle in mode 1 and 5, and another in modes 2 and 4. Because of

the Jordan-Wigner transformation, the fermionic state in (40) corresponds to $|\psi^+_{1,5}\rangle|\psi^+_{2,4}\rangle|0\rangle$ (see Eq.(24)).

However, imperfections in the evolution limit the amount of entanglement between the sites. To measure the effective generated entanglement we use the entanglement fraction and, because of the expected structure of the rainbow state, we focus on $\mathcal{E}_{1,5}$ and $\mathcal{E}_{2,4}$. In order to measure these quantities, we use the entanglement characterisation chip to implement the further transformations $\mathrm{BS}_{15}\,\mathrm{BS}_{24}$, as described in the main text and in the previous section, which interfere the distant modes and allow us to measure entanglement via photodetection in terms of the expectation values

$$N'_m = \langle c^\dagger_m c_m\rangle \ , \quad P'_{nm} = \langle c^\dagger_n c^\dagger_m c_m c_n\rangle \ . \tag{41}$$

The primes in the above equation denotes the fact that the expectation value is taken with respect to the state $|\psi'\rangle = \mathcal{B}|\psi\rangle$, namely after the use of the entanglement characterization chip. Indeed, as we have shown in the previous section, after such transformations the entanglement fraction can be measured in terms of the expectation values (41). Indeed, (33) and (39) read,

$$\mathcal{E}_{1,5} = N'_5 - P'_{51} \ , \quad \mathcal{E}_{2,4} = N'_2 - P'_{24} - P'_{23} + P'_{43} \ . \tag{42}$$

Eqs.(42) give a practical way of evaluating the simulated entanglement fraction from quantities that can be measured experimentally. Finally, also the global fidelity with the rainbow state can be readily obtained from (28) as

$$\mathcal{F} = P'_{25} \ . \tag{43}$$

SUPPLEMENTARY NOTE 5: IMPERFECTIONS

In this section we discuss the role of possible imperfections in tuning the transmission coefficients T_x. From the theoretical point of view, imperfections in T result in a fluctuation of the couplings j_x in the corresponding continuous time quantum walk (13). In the continuos time description, these fluctuations are dynamic, because in the discrete approach the transmission coefficients may be imperfectly tuned in both the spatial directions. In the continuous time version, the corresponding dynamical noise can be modelled as a $j_x(t)$ fluctuating both in x and t. It is known that a possible way for modelling a spin chain with random couplings $j_x(t)$ is via a quantum master equation in the Lindblad form (see, e.g., [7]) where the Lindblad parameters depends on the strength of the noise. In the following we use a similar theoretical approach, where we formally take expectation values over the disorder to obtain an effective (non-unitary) description of the dynamics.

Let us consider the evolution of a state written as a density matrix: $\rho(t+1) = U\,\rho(t)\,U^\dagger$, where $U = \exp\left(-i\hat{P}\sigma^z\right)\exp\left[i\left(\frac{\pi}{2}\mathbb{1} - \hat{\Theta}\right)\sigma^y\right]$ as in Eq.(9) and $T_x = \sin^2\Theta_x$. Let us suppose that the T_x are distributed around the requested value with some probability distribution $D(\hat{T})$. On average, hence, the evolution superoperator can be written in this way.

$$\int D(\hat{T})\;U\,\rho\,U^\dagger = \exp\left(-i\hat{P}\sigma^z\right)\exp\left[i\frac{\pi}{2}\mathbb{1}\,\sigma^y\right]\,e^{\mathcal{L}}[\rho]\;\exp\left[-i\frac{\pi}{2}\mathbb{1}\,\sigma^y\right]\exp\left(+i\hat{P}\sigma^z\right) \ , \tag{44}$$

where

$$e^{\mathcal{L}}[\rho] = \int D(\hat{T})\;e^{\left[-i\arcsin\left(\sqrt{\hat{T}}\right)\,\sigma^y\right]}\,\rho\,e^{\left[i\arcsin\left(\sqrt{\hat{T}}\right)\,\sigma^y\right]} \ . \tag{45}$$

It turns out that the superoperator $\mathcal{L}$ takes the form

$$\mathcal{L}[\rho] = -I(\hat{\Phi}\sigma^y \rho - \rho\,\hat{\Phi}\sigma^y) + \left(\hat{\Lambda}^{\frac{1}{2}}\sigma^y \rho\,\sigma^y \hat{\Lambda}^{\frac{1}{2}} - \frac{1}{2}\{\hat{\Lambda}, \rho\}\right) \ , \tag{46}$$

with some parameters $\hat{\Phi}$ and $\hat{\Lambda}$ that depend on the distribution of the $D(\hat{T})$. In the following we consider relevant noise distributions and show that the average effect of dynamical noise is expected not to alter significantly the results.

- **Uniform distribution**: Here we assume that $T_x = \sin^2(\epsilon j_x)(1+\sigma\eta)$, where η is uniformly distributed in $\left[-\frac{1}{2}, \frac{1}{2}\right]$. In the above equation σ is the relative strength of the disorder and j_x is the coupling that we want to implement. Owing to some lengthy calculations, one can show that for $(\epsilon, \sigma) \to (0,0)$ the result is

$$\Phi_x \approx \epsilon\left(\frac{j_x}{2} - \frac{j_x}{192}\sigma^2\right) \ , \qquad \Lambda_x \approx \frac{j_x^2}{192}\sigma^2\epsilon^2 \ . \tag{47}$$

  As $\Lambda_x = \mathcal{O}(\epsilon^2)$, and because of the small factor 1/192, the effect of the dynamical noise is expected to alter very little the results predicted by the (13), as long as σ is small.

- **Gaussian distribution**: Here we assume that $T_x = \sin^2(\epsilon j_x)(1+\eta)$, where η is distributed according to a Gaussian distribution centered around 0 with variance σ. In this case

$$\Phi_x \approx \epsilon\left(\frac{j_x}{2} - \frac{j_x}{64}\sigma^2\right) \ , \qquad \Lambda_x \approx \frac{j_x^2}{64}\sigma^2\epsilon^2 \ . \tag{48}$$

  We obtain again an effective unitary continuous quantum walk with renormalized couplings. Although the coefficient 1/64 is a bit higher than before, one can see that the effect of noise adds only a small perturbation to the predicions of (13).

The predicted stability is verified with a numerical analysis in Supplementary Fig. 5. One can see that when $M \gg 1$, the dynamical errors plays basically no role even for large values of σ. On the other hand, for the experimental setting where $M = 6$ one can see that the expected correlation pattern of the states (40) is maintained when $\sigma = 0.2$, but is severely changed when $\sigma = 1$.

To better understand how the error scales as a function of the system size we decompose the operator $\mathcal{L}$ in (46) as

$$\mathcal{L} = \epsilon\mathcal{H}_0 + \sigma^2\epsilon^2\mathcal{E} \ , \tag{49}$$

where $\mathcal{H}_0$ is the error-free operator while $\mathcal{E}$ is the source of errors. We assume that the main source of errors is due to the operators Λ_x, since Φ_x can be changed even in the imperfect case to force $\Phi_x = \epsilon j_x/2$. The value of ϵ is fixed in order to keep the discretization error down to the desired accuracy after the M steps. The effect of imperfections after the M steps can be estimated from the trace distance between the error-free evolution and the expected one when $\sigma \neq 0$

$$\|\mathrm{e}^{2t^*\mathcal{H}_0} - \mathrm{e}^{M\mathcal{L}}\| \simeq \|\mathrm{e}^{N\mathcal{H}_0} - \mathrm{e}^{N\mathcal{H}_0 + N\epsilon\sigma^2\mathcal{E}}\| \simeq N\epsilon\sigma^2 \|\int_0^1 ds\, \mathrm{e}^{N(1-s)\mathcal{H}_0}\mathcal{E}\mathrm{e}^{Ns\mathcal{H}_0}\| \tag{50}$$

$$= \mathcal{O}(N\epsilon\sigma^2) = \mathcal{O}(N^2\sigma^2/M), \tag{51}$$

where we used (17), $N+1 \approx N$ for large N, and the first order expansion [8] in terms of $\epsilon\sigma^2$, since $\epsilon\sigma^2 \ll 1$. Therefore, while in the error-free case ϵ was chosen in order to minimize the discretization error (thus forcing $M \gg N$), in the presence of imperfections, namely when $\sigma \neq 0$, one has to satisfy also the constraint $\epsilon \ll (N\sigma^2)^{-1}$, or alternatively $M \gg N^2\sigma^2$, to minimize long-time errors due to imperfect fabrication.

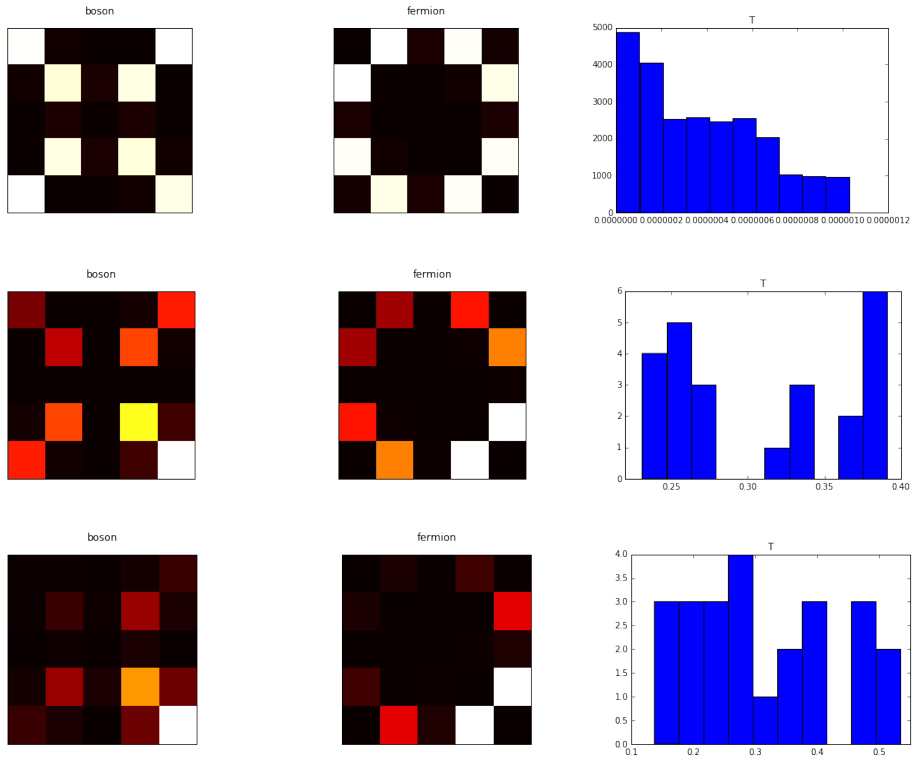

Supplementary Figure 5. Correlation matrix $|\langle c_i^\dagger c_j\rangle|$, for $i, j = 1, \ldots 5$ after the quantum transport chip, and distribution of the transmissivities. The expected states for bosonic and fermionic initial states are written in Eq.(40). In the plots in the first and second column the colors range between zero (no correlations) and white (maximal correlations). a) $M = 6000$ and $\sigma = 4$, b) $M = 6$ and $\sigma = 0.2$, c) $M = 6$ and $\sigma = 1$ for a uniform distribution.

SUPPLEMENTARY NOTE 6: PHOTON SOURCE CHARACTERISATION

For this experiment, a mode-locked Ti:Sapphire laser with central wavelength of 785 nm, 160 fs pulse width and 76 MHz repetition rate is used as pump. Second harmonic generation (SHG) in a beta barium borate (BBO) crystal is exploited for conversion into 392.5 nm wavelength. The generation of single-photon pairs is achieved via Spontaneous Parametric Down Conversion (SPDC) by sending the converted pulses through a 2 mm-thick BBO crystal. The generated photons are spectrally filtered by means of interferential filters with 3nm bandwidth, centered at 785 nm. Typical detection rates of generated photons were approximately $\sim$ 120 KHz for singles and $\sim$ 7 KHz for pairs. Schematics of the source is shown in Supplementary Fig. 6. The unconverted residual beam at 785 nm from the SHG is separated through a dichroic mirror and used as simulation beam for alignment and classical characterisation of the integrated device.

The generated photon state is in the general entangled form $|\Psi_{2\mathrm{p}}^{\chi}\rangle = 2^{-1/2}(|\mathrm{HV}\rangle + e^{\imath\chi}|\mathrm{VH}\rangle)$, where the phase χ depends on the angle of the crystal's optical axis. Due to birefringence and dispersion effects in the SPDC BBO crystal, a walk-off compensation between the two polarizations

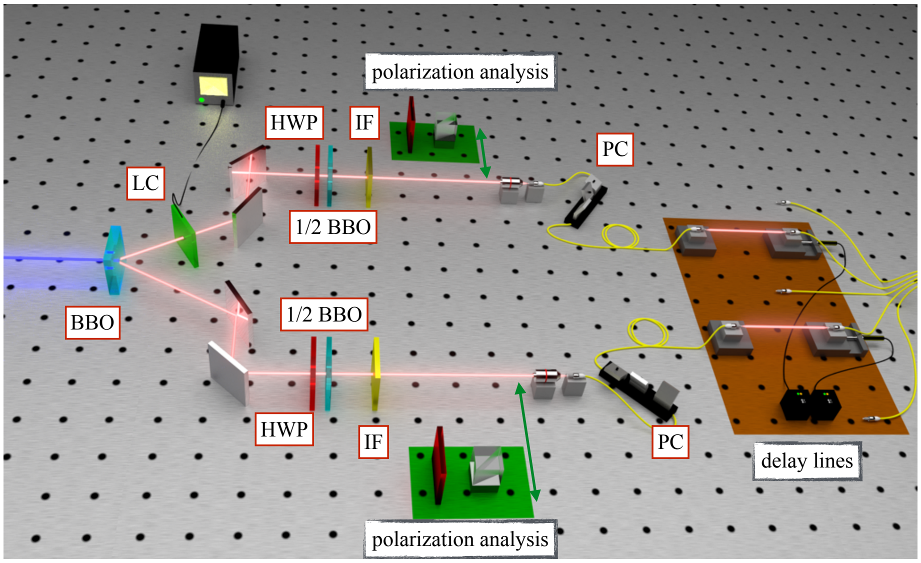

Supplementary Figure 6. Visual schematics of the single-photon source used for the experiment. BBO: beta barium borate crystal used for parametric down conversion; LC: liquid crystal for entanglement phase χ control; HWP & 1/2 BBO: half-waveplate and half beta barium borate crystal for walk-off compensation; PC: polarization compensation. The polarization analysis is comprised of half-waveplate and polarizing beam splitter. The delay line is used to temporally synchronize the photon and couple them into the single mode fiber array.

is required for full indistinguishability. This is done by using a combination of an half-wave plate and a 1mm thick BBO crystal in each of the two photons' paths.

A controlled liquid crystal retarder is used in one arm to vary the phase χ, allowing to attain the required states. Half-wave plates and polarizing beamsplitters at each arm can be inserted when necessary to perform polarization analysis. The photons are then collected into single-mode optical fibers mounted on 3-paddle polarization controllers, used to compensate the polarization due to the bending in the fibers, and sent through delay lines to achieve temporal synchronization of the photons. Indistinguishability of the photon source is measured by performing an Hong-Ou-Mandel (HOM) two photon interference [9] interference experiment in a 50/50 beam splitter (BS) after polarization analysis. The obtained visibilities are $V_{\rm raw}^{\rm source} = -0.914 \pm 0.002$ for raw data and $V_{\rm corr}^{\rm source} = -0.95 \pm 0.01$ corrected for accidental counts (see Supplementary Fig. 7a). Similar interference measurements have been performed for the two entangled states $|\Psi_{2\rm p}^{+}\rangle$ and $|\Psi_{2\rm p}^{-}\rangle$ to characterize the quality of the generated entangled. The observed visibilities where $V_{\rm raw}^{\rm B} = -0.880 \pm 0.002$ and $V_{\rm raw}^{\rm F} = 0.90 \pm 0.01$ for raw measurements, and $V_{\rm corr}^{\rm B} = -0.957 \pm 0.002$ and $V_{\rm corr}^{\rm F} = 0.97 \pm 0.01$ corrected for accidental counts (see Supplementary Fig. 7b). Noise introduced by multi-photon emission terms for the adopted source correspond to a level of $\sim 1.5\%$, and are thus negligible with respect to other imperfections.

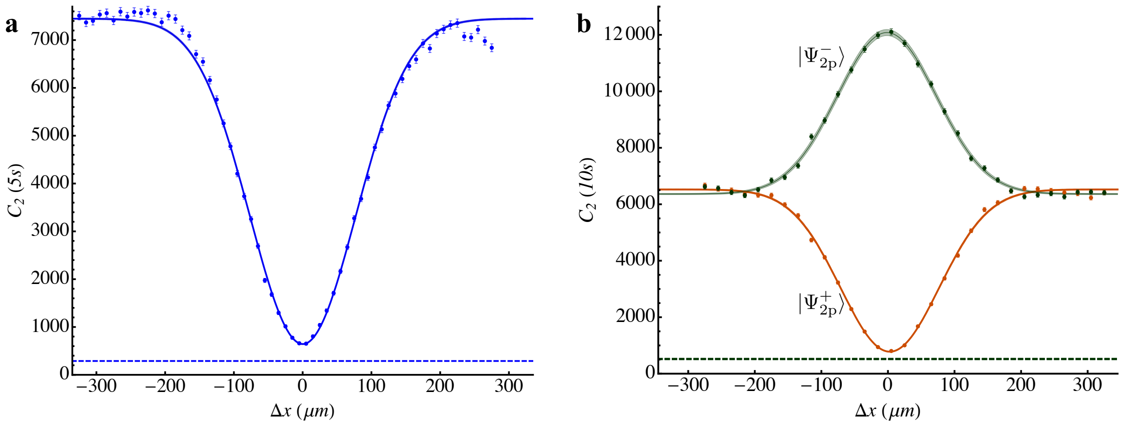

Supplementary Figure 7. **a.** Hong-Ou-Mandel interference scan over 600 μm of two indistinguishable photons. **b.** Hong-Ou-Mandel interference dip for state $|\Psi^+_{2p}\rangle$ (orange) and peak for state $|\Psi^-_{2p}\rangle$ (green). Thick lines: best fit curves. Shaded regions: 1σ intervals for the best fit curves. Dashed lines: level for accidental coincidences.

SUPPLEMENTARY NOTE 7: MODELING THE EVOLUTION WITH THE RECONSTRUCTED UNITARY TRANSFORMATION

Here we briefly discuss how to model the evolution of a polarization entangled state through the quantum transport interferometer. We consider the evolution of an entangled pair through a unitary operator U^π, which in this case presents a small difference for the two orthogonal polarizations. The action of U^π on the field operator before $(a^\dagger_{j,\pi})$ and after $(b^\dagger_{i,\pi})$ the evolution, where $\pi = \mathrm{H}, \mathrm{V}$ labels the polarization state, is expressed by $a^\dagger_{j,\pi} = \sum_i U^\pi_{j,i} b^\dagger_{i,\pi}$. The input state of the process is a two-photon entangled pair in the $|\Psi^\pm_{2p}\rangle_{i,j}$ Bell state, where the photons are injected in input ports i and j:

$$|\Psi^\pm_{2\mathrm{p}}\rangle_{i,j} = \frac{1}{\sqrt{2}}(|\mathrm{H}\rangle_i|\mathrm{V}\rangle_i \pm |\mathrm{V}\rangle_i|\mathrm{H}\rangle_j) = \frac{1}{\sqrt{2}}(a^\dagger_{i,\mathrm{H}}a^\dagger_{j,\mathrm{V}} \pm a^\dagger_{i,\mathrm{V}}a^\dagger_{j,\mathrm{H}})|0\rangle. \tag{52}$$

The state after the evolution can be written as:

$$\begin{aligned}|\Psi^\pm_{2\mathrm{p}}\rangle_{i,j} \overset{U^\pi}{\rightarrow} \frac{1}{\sqrt{2}}\left[\left(\sum_m U^\mathrm{H}_{i,m}b^\dagger_{m,\mathrm{H}}\right)\left(\sum_n U^\mathrm{V}_{j,n}b^\dagger_{n,\mathrm{V}}\right) \pm \left(\sum_n U^\mathrm{V}_{i,n}b^\dagger_{n,\mathrm{V}}\right)\left(\sum_m U^\mathrm{H}_{j,m}b^\dagger_{m,\mathrm{H}}\right)\right]|0\rangle = \\ = \frac{1}{\sqrt{2}}\left[\sum_{m,n}\left(U^\mathrm{H}_{i,m}U^\mathrm{V}_{j,n} \pm U^\mathrm{H}_{j,m}U^\mathrm{V}_{i,n}\right)b^\dagger_{m,\mathrm{H}}b^\dagger_{n,\mathrm{V}}\right]|0\rangle.\end{aligned} \tag{53}$$

The transition amplitudes from input state $|\Psi^\pm_{2p}\rangle_{i,j}$ to the output configurations $|r\mathrm{H}, s\mathrm{V}\rangle$ and $|s\mathrm{H}, r\mathrm{V}\rangle$ for $r \neq s$ are respectively:

$$\langle r\mathrm{H}, s\mathrm{V}|U|\Psi^\pm_{2\mathrm{p}}\rangle_{i,j} = \frac{1}{\sqrt{2}}\left(U^\mathrm{H}_{i,r}U^\mathrm{V}_{j,s} \pm U^\mathrm{H}_{j,r}U^\mathrm{V}_{i,s}\right) \tag{54}$$

$$\langle s\mathrm{H}, r\mathrm{V}|U|\Psi^\pm_{2\mathrm{p}}\rangle_{i,j} = \frac{1}{\sqrt{2}}\left(U^\mathrm{H}_{i,s}U^\mathrm{V}_{j,r} \pm U^\mathrm{H}_{j,s}U^\mathrm{V}_{i,r}\right) \tag{56}$$

where the $\pm$ signs in the transition amplitudes depend on the symmetry of the input entangled state. We can define the submatrices $U^{i,j}_{r,l}$ of U^{H} and U^{V} as:

$$U^{i,j}_{r,s} = \begin{pmatrix} U^{\mathrm{H}}_{i,r} & U^{\mathrm{H}}_{j,r} \\ U^{\mathrm{V}}_{i,s} & U^{\mathrm{V}}_{j,s} \end{pmatrix}. \tag{57}$$

If the input state is in the singlet anti-symmetric state $|\Psi^-_{2\mathrm{p}}\rangle_{i,j}$, the probability of obtaining a photon on modes r and s can be expressed as:

$$\mathrm{Prob}(r,s|\Psi^-_{i,j}) = \frac{1}{2}\left[\left|\det\left(U^{i,j}_{r,s}\right)\right|^2 + \left|\det\left(U^{i,j}_{s,r}\right)\right|^2\right], \tag{58}$$

$$\mathrm{Prob}(r,r|\Psi^-_{i,j}) = \frac{1}{2}\left|\det\left(U^{i,j}_{r,r}\right)\right|^2 \tag{60}$$

thus depending on the determinants of the submatrices $U^{i,j}_{r,s}$. Conversely, if the input state is symmetric $|\Psi^+_{2\mathrm{p}}\rangle_{i,j}$ we obtain:

$$\mathrm{Prob}(r,s|\Psi^+_{i,j}) = \frac{1}{2}\left[\left|\mathrm{per}\left(U^{i,j}_{r,s}\right)\right|^2 + \left|\mathrm{per}\left(U^{i,j}_{s,r}\right)\right|^2\right], \tag{61}$$

$$\mathrm{Prob}(r,r|\Psi^+_{i,j}) = \frac{1}{2}\left|\mathrm{per}\left(U^{i,j}_{r,r}\right)\right|^2 \tag{63}$$

thus depending on permanents of the submatrices $U^{i,j}_{r,s}$.

SUPPLEMENTARY NOTE 8: CHARACTERISATION OF THE QTC DEVICE

The generated photons are coupled into the integrated device using a single-mode fiber array, and collected after evolution through the interferometer using a multimode fiber array which sends the photons to Single Photon Avalanche Photodiodes (SPADs). Both input and output fiber arrays are mounted on roto-translational stages. The alignment is performed using the unconverted residual from the SHG. Overall transmission efficiency from delay lines to the photodetectors with the first chip is approximately 12%, which includes transmission losses inside the chip and coupling losses at the three interfaces between delay line, input fiber array, chip and output fiber array. The phase χ of the entangled state is set by exploiting HOM interference for the output bunching contributions. This is obtained by inserting an additional in-fiber BS at output mode 1 and by measuring the output two-fold coincidences. By maximizing the coincidence rate we have a symmetric (bosonic) state $|\Psi^+_{2\mathrm{p}}\rangle$ and by minimizing it we have an anti-symmetric (fermionic) state $|\Psi^-_{2\mathrm{p}}\rangle$.

Photon pairs are sent into the device to simulate bosonic and fermionic transport in the lattice, respectively exploiting the symmetric and anti-symmetric nature of the Bell states $|\Psi^+_{2\mathrm{p}}\rangle$ and $|\Psi^-_{2\mathrm{p}}\rangle$. The simulation of the transport of a spin state in a 1D chain, initially in a Néel's state $|\downarrow\uparrow\downarrow\uparrow\downarrow\rangle$, is obtained by injecting the $|\Psi^-_{2\mathrm{p}}\rangle$ state in input 2 and 4. Switching from one state to the other is achieved by changing the phase χ with the liquid crystal retarder. Detection of output states in which two photons are in the same spatial mode is obtained by inserting an in-fiber 50/50 beam splitter to the chip's output.

Tomography of the QTC device

The reconstruction of the unitary matrix which describes the actual interferometer corresponds to retrieving the values of its elements (moduli and complex phases). Unitary's moduli are sufficient

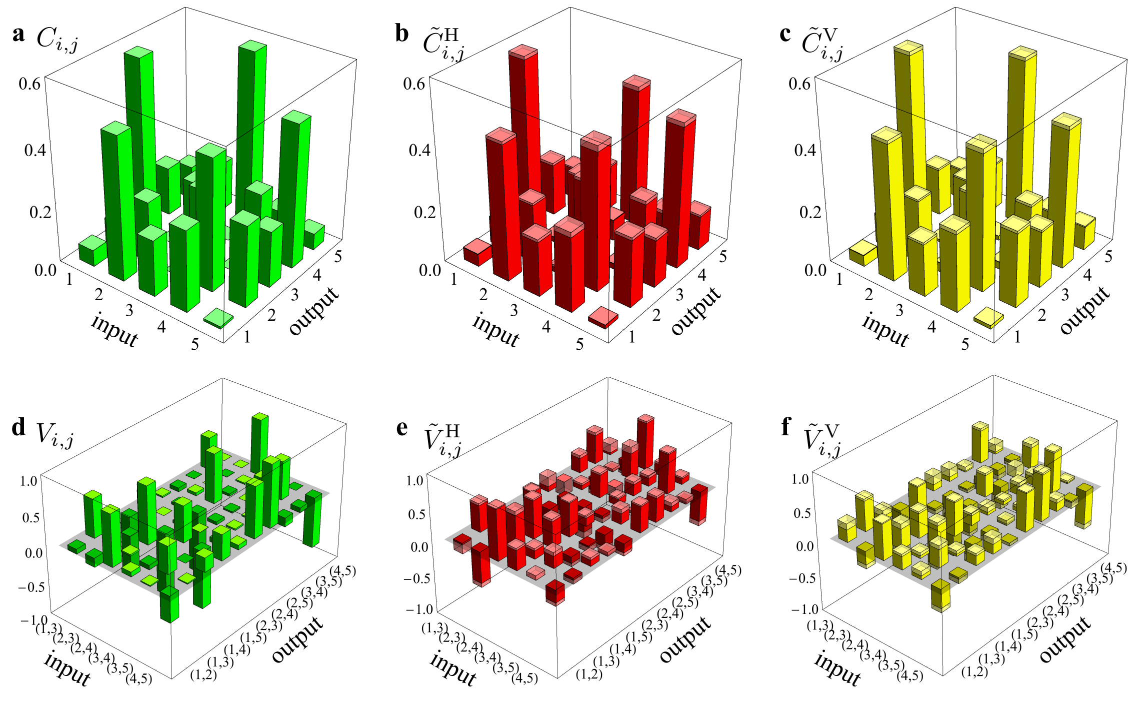

Supplementary Figure 8. Power-splitting ratios with classical light (**a-c**) and two-photon interference dips and peaks visibilities (**d-f**) results: **a-d** calculations from the theoretical unitary U, **b-e** experimental results with horizontal polarization and **c-f** experimental results with vertical polarization. Lighter regions are 1σ experimental errors.

to describe single-particle and classical behavior, while phase terms are crucial for two-particle interference. The unitary matrix is experimentally reconstructed by measuring the single-photon output distributions (or the equivalent power splitting ratios with classical light) for all inputs and two photon interference for several pairs of inputs [10,11]. More specifically, we measured the power splitting ratios with classical light and two-photon Hong-Ou-Mandel visibilities for 6 different input states for both polarizations H and V (see Supplementary Fig. 8). Characterisation of the device is performed starting from the chip structure of Fig. 1b and by assuming unknown values for all the fabrication parameters (directional couplers trasmittivities and phases between the modes). The value of the parameters are obtained by minimizing a suitable χ-square function, while errors in the reconstruction are retrieved with a Monte Carlo simulation starting from the experimental power-splitting ratios and two-photon data [11].

The fidelity of the reconstructed unitary matrix, compared to the theoretical model, is defined as $\mathcal{F}^{\pi} = \frac{1}{5}|\mathrm{Tr}[(U^{\dagger}\tilde{U}^{\pi})]|$ with $\pi = \mathrm{H}, \mathrm{V}$. The obtained fidelities for the two polarizations with respect to the theoretical model are $\mathcal{F}^{\mathrm{H}} = 0.962 \pm 0.001$ and $\mathcal{F}^{\mathrm{V}} = 0.977 \pm 0.002$. The polarization insensitivity of the device is confirmed by the results for the fidelity between the reconstructed unitary in H and V polarization of $\mathcal{F}^{\mathrm{H/V}}_{\mathrm{Reco}} = 0.99 \pm 0.01$ (see Supplementary Fig. 9).

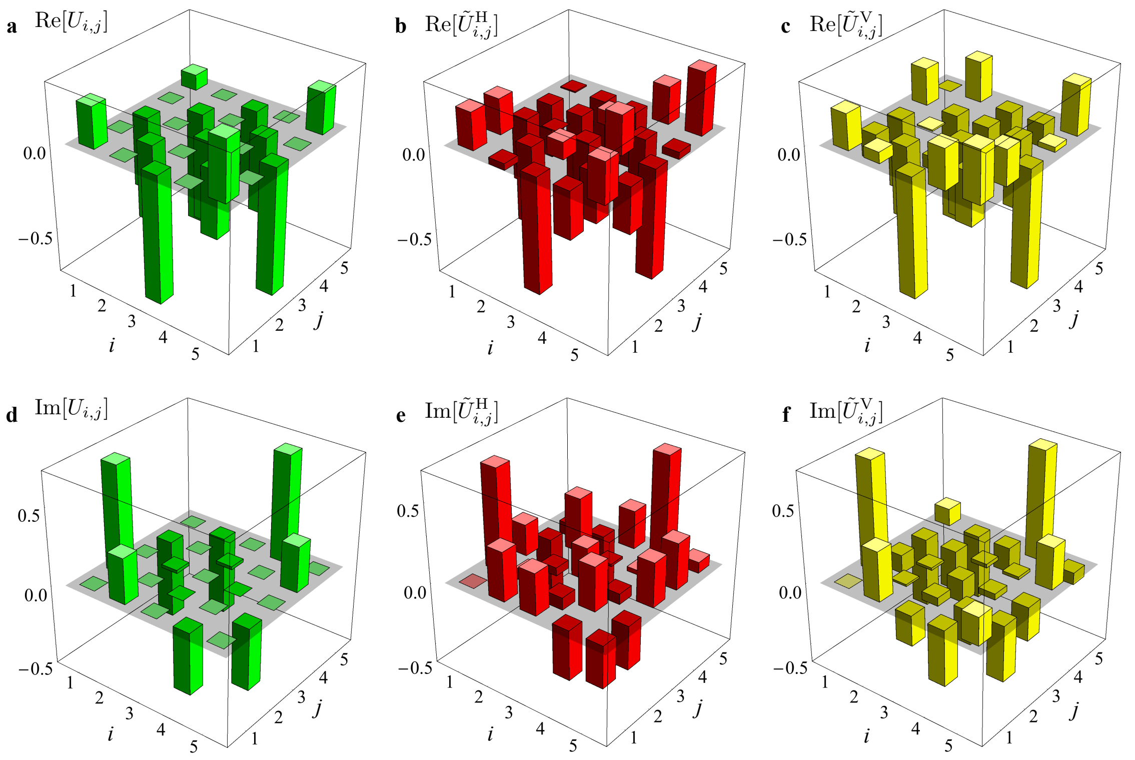

Supplementary Figure 9. Real and imaginary terms for **a-d** theoretical unitary matrix, **b-e** reconstructed unitary matrix for horizontal polarization, and **c-f** reconstructed unitary matrix for vertical polarization.

Single- and two-photon measurements.

Experimental single- and two-photon distributions, measured in both polarizations H and V, further confirm the polarization insensitivity (see Supplementary Fig. 10). A comparison between the experimental data with entangled input states and the predictions obtained from the theoretical unitary and the ones from the characterized unitary is finally shown in Supplementary Fig. 11.

SUPPLEMENTARY NOTE 9: CHARACTERISATION OF THE ECC DEVICE

For an ideal fermionic quantum spin transport, the target state would be of the form $|\psi_{\mathrm{ideal}}\rangle = |\psi^+_{1\mathrm{p}}\rangle_{24}|\psi^+_{1\mathrm{p}}\rangle_{15}|0\rangle_3$. Our device is a finite engineered chain and the generated the state is $|\psi_{\mathrm{out}}\rangle = (\alpha|10\rangle_{15} + \beta|01\rangle_{15})(\gamma|10\rangle_{24} + \delta|01\rangle_{24})|0\rangle_3$, where the terms α, β, γ and δ define an unbalancement due to the approximation of the perfect state.

The coherence between the path entangled states is studied using the second device, comprised of integrated beam splitters and thermal phase shifters. In particular, with suitable phases before the two beam-splitters, the target state would result in a photon in output mode 2 and a photon in output mode 5. Before performing the final measurement, the phase χ of the liquid crystal has

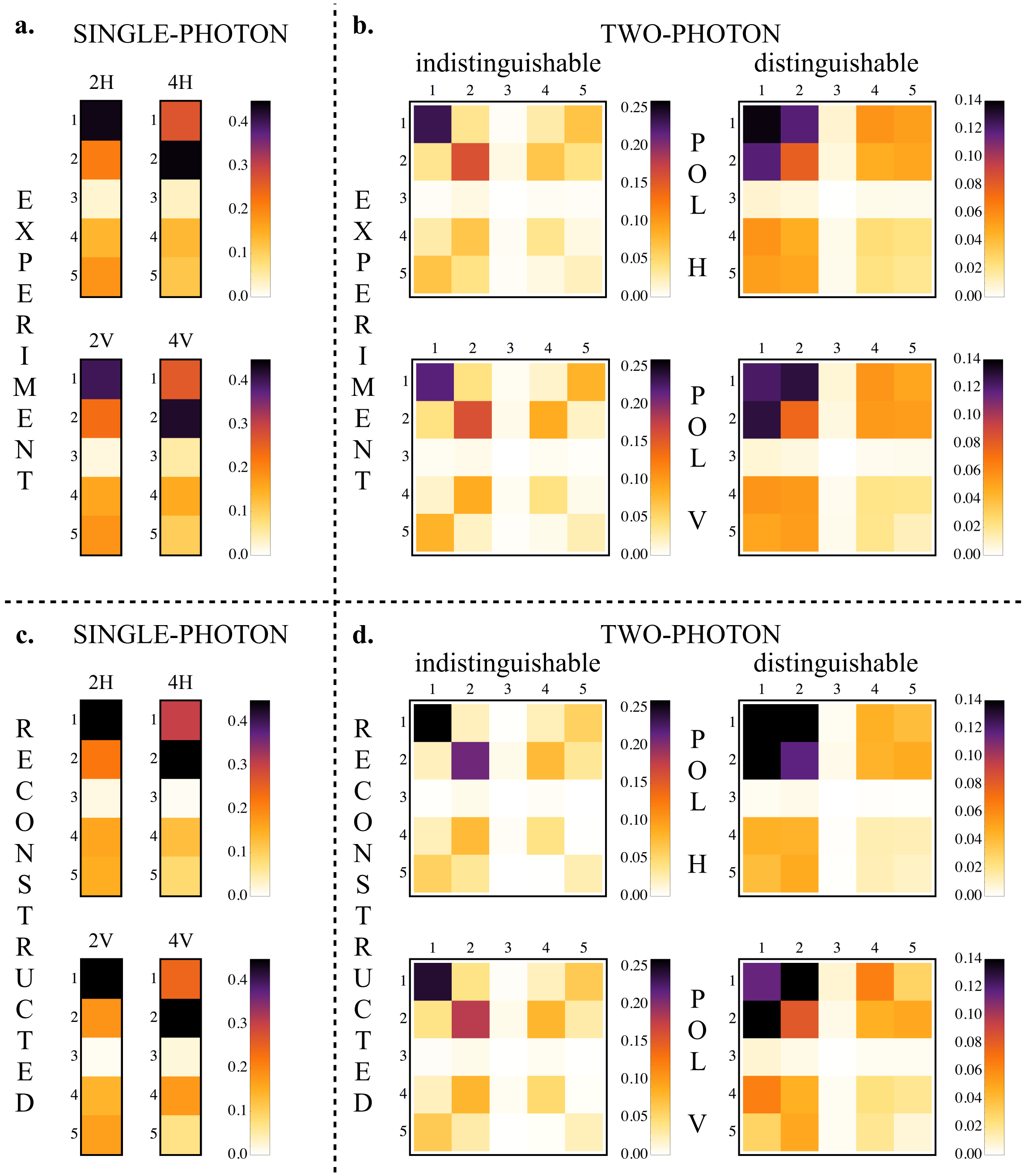

Supplementary Figure 10. **a-b.** Experimental single-photon couplings for input 2 and 4 and two photon unitary transformation, both indistinguishable and distinguishable, for polarization H and V. **c-d.** Numerical prediction from experimentally reconstructed unitary transformation of single-photon probabilities for input 2 and 4 and two-photon interference, both indistinguishable and distinguishable, distribution for polarization H and V. Both single- and two-photon distributions show the polarization insensitivity of the device.

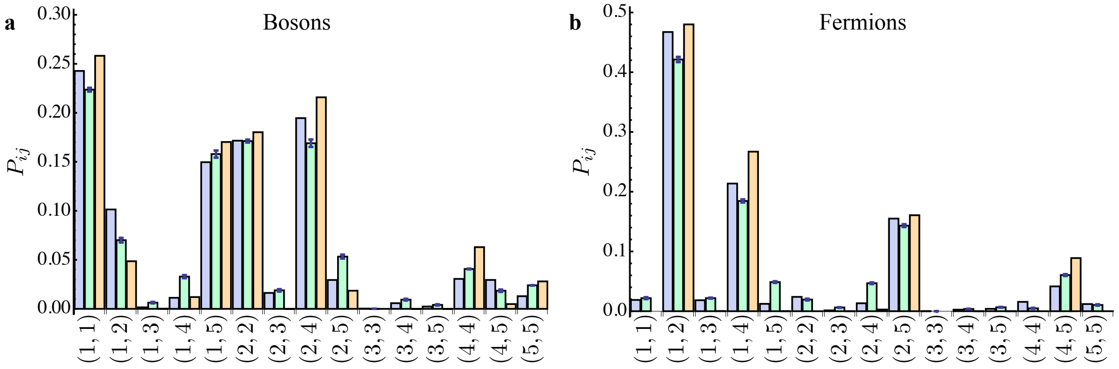

Supplementary Figure 11. Comparison between experimental data (green bars), predictions obtained from the ideal unitary (orange bars) and from the reconstructed one (blue bars). **a**, Two-photon probability distributions for the bosonic case and **b**, fermionic case.

to be set in order to inject the correct anti-symmetric state. This procedure is necessary since the single-mode fibers introduce an additional phase χ_{fiber} that has to be compensated. The singlet $|\Psi^-_{2\mathrm{p}}\rangle$ state is set by measuring the physical quantity $G = (P'_{12} + P'_{14} + P'_{25} + P'_{45}) - (P'_{24} + P'_{15})$ at the output of the second device, where P'_{ij} is the coincidence probability at modes i and j. Such quantity is maximized for input state $|\Psi^-_{2\mathrm{p}}\rangle$ and minimized for input state $|\Psi^+_{2\mathrm{p}}\rangle$, and is independent from the tunable phase ϕ_2 and ϕ_5.

The phase shifters in the second device are actively controlled via thermal resistors (see Supplementary Fig. 12). Each thermal resistor is connected to an external power supply with independent voltage channel control and the applied voltage ranges between 0 V to 7 V, in which a full 2π oscillation period is seen. Overall transmission efficiency from delay lines to the photodetectors is approximately 0.6%, which includes transmission of the two chips (25% for the first one and 40% for the second one) and coupling efficiency at the four interfaces between delay line, input fiber array, first chip, second chip and output fiber array. This value can be improved by using for instance index matching fluid between the two devices and more precise micromanipulation stages for the alignment of the different components. Coincidences for all outputs are measured by singularly tuning the applied phase shift from each thermal resistors, showing a 2π oscillation within ~ 0.8 W dissipated power. When evaluating the expected curves for the quantities S_1 and S_5 as a function of ϕ_5, losses corresponding to an additional efficiency $\eta_5 = 0.36$ is considered for mode 5 at the interface between the two devices. This quantity is retrieved from characterisation with classical light.

SUPPLEMENTARY NOTE 10: DETECTING BLOCK ENTANGLEMENT VIA THE VON NEUMANN ENTROPY

Another fundamental quantity for the study of quantum quenches is the entanglement entropy for a particular bipartition of the chain. This quantity is defined as the von Neumann entropy $S(\rho) = -\operatorname{Tr}[\rho \log_2 \rho]$ of the reduced state of either part of the chain. In the typical scenario the chain is divided in two parts A and B, where A is the first half and B is the second half of the chain. The entanglement entropy is $S_{\mathrm{A,B}} = S(\rho_{\mathrm{A}}) = S(\rho_{\mathrm{B}})$ being ρ_{A} and ρ_{B} the reduced density

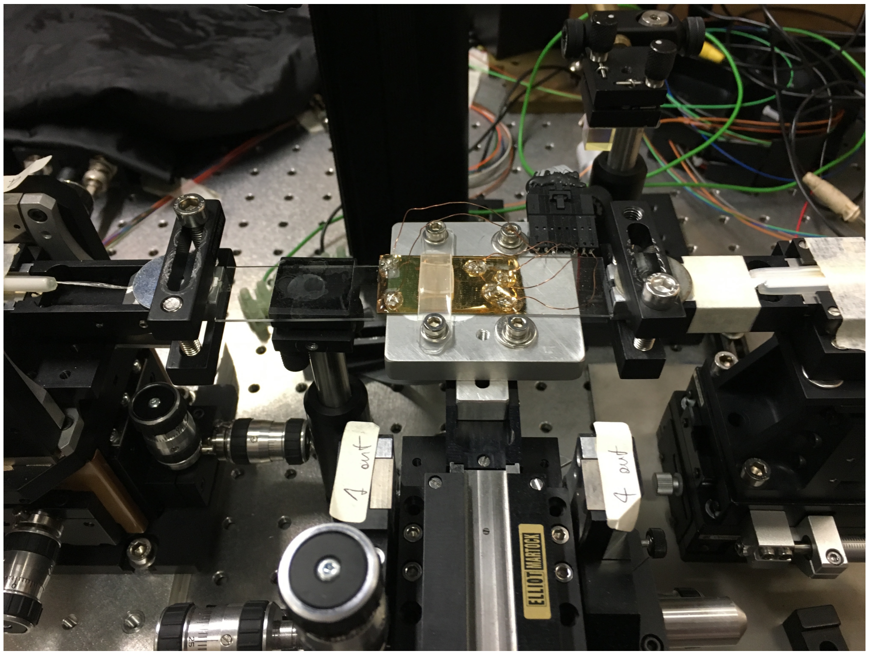

Supplementary Figure 12. Photograph of the experimental setup. From the left: input single-mode fiber array, chip with quantum transport device, chip with thermal resistor and entanglement check device, output multi-mode fiber array.

matrices of the left and right side of the chain state [12]. A volume law of entanglement corresponds to a scaling $S \propto N$ as a function of the length of the chain. By construction a rainbow state of $N = 2L+1$ spins or $N = 2L$ spins has the maximal amount of entanglement entropy $S_{\mathrm{A,B}} = L \propto N$. The entanglement entropy however is not defined only in terms of pairwise entanglement between *pairs* of spins in the two bipartitions, but can also detect block forms of entanglement. Because of its importance we now show how it can be measured.

When the initial state (4) satisfies the Wick's theorem, then there is a simple expression [12] in terms of the eigenvalues ζ_j of the reduced $L \times L$ matrix $C_{ij} = \langle c_i^\dagger c_j \rangle$ where $i, j = 1, \ldots, L$:

$$S_{\mathrm{A,B}} = \sum_{j=1}^{L} \left[-\zeta_j \log_2 \zeta_j - (1-\zeta_j) \log_2 (1-\zeta_j) \right] \ . \tag{64}$$

Some of the elements of the reduced covariance matrix C can be obtained without the help of the entanglement characterization chip via the measurement of

$$N_m = \langle c_m^\dagger c_m \rangle \ , \qquad\qquad P_{nm} = \langle c_n^\dagger c_m^\dagger c_m c_n \rangle \ . \tag{65}$$

Indeed, clearly $C_{mm} = N_m$ and from Wick's theorem $|C_{mn}|^2 = N_n N_m - P_{nm}$, so the only missing element is the phase of the off-diagonal elements C_{nm}. The phase of each pair can be measured via extra beam splitter devices like the entanglement characterization chip. Indeed, applying a beam

splitter BS_{nm} and measuring the expected number of particles one finds that

$$\langle \mathrm{BS}^\dagger_{nm} c^\dagger_n c_n \mathrm{BS}_{nm} \rangle = \frac{C_{nn} + C_{mm} - 2\Re[C_{nm}]}{2} \ , \tag{66}$$

$$\langle \mathrm{BS}^\dagger_{nm} c^\dagger_m c_m \mathrm{BS}_{nm} \rangle = \frac{C_{nn} + C_{mm} + 2\Re[C_{nm}]}{2} \ . \tag{67}$$

Similarly, by applying BS_{nm} together with a $\pi/4$ phase shift F_n on mode n one finds

$$\langle F^\dagger_n \mathrm{BS}^\dagger_{nm} c^\dagger_n c_n \mathrm{BS}_{nm} F_n \rangle = \frac{C_{nn} + C_{mm} + 2\Im[C_{nm}]}{2} \ , \tag{68}$$

$$\langle F^\dagger_n \mathrm{BS}^\dagger_{nm} c^\dagger_m c_m \mathrm{BS}_{nm} F_n \rangle = \frac{C_{nn} + C_{mm} - 2\Im[C_{nm}]}{2} \ . \tag{69}$$

Using the above operations and measurements then one can extract both the real and imaginary parts of C_{nm}.

When $L = 2$ these extra operations are not needed, and only the measurements (65) suffice to estimate the entropy (64). Indeed, when $L = 2$ the eigenvalues of the reduced correlation matrix are

$$\zeta_{1,2} = \frac{C_{11} + C_{22} \pm \sqrt{(C_{11} - C_{22})^2 + 4|C_{12}|^2}}{2} \tag{70}$$

$$= \frac{N_1 + N_2 \pm \sqrt{(N_1 + N_2)^2 - 4P_{12}}}{2} \ , \tag{71}$$

which can be directly computed from the measurement of (65) without extra entanglement characterization chips.

To evaluate the entanglement entropies we restrict the output probability distribution to the collision-free space, thus post-selecting the events in which the two photons exit from different output ports. The average number of photons $\tilde{N}_i$ can be retrieved from the measured two-photon distribution $\tilde{P}_{i,j}$ as:

$$\tilde{N}_i = \sum_m (1 - \delta_{i,m}) \tilde{P}_{i,m}. \tag{72}$$

The number of photons $\tilde{N}_i$ satisfies the constraint $\sum_{j=1}^{5} \tilde{N}_j = 2$. The obtained experimental entanglement entropy is then $S^{\mathrm{exp}}_{12,345} = 1.63 \pm 0.06$. To understand the significance of this value, different comparisons can be performed. A perfect state transfer Hamiltonian at half transfer time would lead to an entropy $S^{\mathrm{PST}}_{12,345} = 2$. The expected value for the QTC with the theoretical unitary calculated from the fabrication parameters is $S^{\mathrm{T}}_{12,345} \sim 1.75$. Finally, we observe that the measured $S^{\mathrm{exp}}_{12,345}$ is compatible with the expected value calculated with the reconstructed QTC transformations $\{\tilde{U}^{\mathrm{H}}, \tilde{U}^{\mathrm{V}}\}$, which reads $S^{\mathrm{R}}_{12,345} = 1.52 \pm 0.06$. The error bar in this last parameter is due to the error in the reconstruction process.

Similarly, the entanglement entropy $S_{123,45}$ is obtained from Eqs. (64) and (71) by replacing modes $(1, 2)$ with modes $(4, 5)$. The obtained experimental entanglement entropy is $S^{\mathrm{exp}}_{123,45} = 1.446 \pm 0.014$. Again, this has to be compared with $S^{\mathrm{PST}}_{123,45} \sim 2$ (perfect state transfer), $S^{\mathrm{T}}_{123,45} \sim 1.75$ (theoretical QTC unitary with fabrication parameters) and $S^{\mathrm{R}}_{123,45} = 1.47 \pm 0.07$ (reconstructed transformations $\{\tilde{U}^{\mathrm{H}}, \tilde{U}^{\mathrm{V}}\}$).

SUPPLEMENTARY NOTE 11: EVALUATING THE ENTANGLEMENT OF THE QTC'S OUTPUT VIA MAXIMUM LIKELIHOOD ESTIMATION

Relevant quantities to evaluate the purity and the amount of entanglement of the QTC's output can be retrieved from the state correlation matrix $C_{i,j} = \langle c_i^\dagger c_j \rangle$. To this end, we estimated an equivalent set of correlation matrices, satisfying all properties of a two-fermion physical state, that better describes the measured data. The diagonal elements of the correlation matrix $C_{i,i} = N_i = \langle c_i^\dagger c_i \rangle$ and the moduli of the off-diagonal elements $|C_{i,j}| = (N_i N_j - P_{i,j})^{1/2}$ can be directly measured without additional interferometers at the output of the QTC. The complex phases of the off-diagonal terms of the $C_{i,j}$ matrix need to be evaluated to retrieve the eigenvalues ζ_k of $C_{i,j}$. The ECC chip permits to obtain information on the phases of $C_{1,5}$ and $C_{2,4}$. Indeed, by measuring the output of the ECC chip it can be shown that $N_5' - N_1' = 2\text{Re}(C_{1,5})$ and $N_2' - N_4' = 2\text{Re}(C_{2,4})$, where the phases ϕ_2 and ϕ_5 of the ECC are set so as to maximize $P_{2,5}'$. This additional measurement, combined with the knowledge of $|C_{1,5}|$ and $|C_{2,4}|$, provides information on the complex phases of $C_{1,5}$ and $C_{2,4}$ up to a sign in the imaginary part. Given the available information on the $|C_{i,j}|$ and on the real parts of $C_{1,5}$ and $C_{2,4}$, one can obtain a lower and upper bound on the actual purity of the measured state. This could be done in principle by replacing the moduli with the experimentally estimated values and by exploiting the available partial information on the phase of $C_{1,5}$ and $C_{2,4}$. The remaining unknown complex phases of $C_{i,j}$ and the sign of $\text{Im}(C_{1,5})$ and $\text{Im}(C_{2,4})$ can be left as free parameters to search numerically the minimum and maximum of the purity given the available information. However, such a direct approach does not provide a meaningful bound on the purity, since the properties of a physical correlation matrix $C_{i,j}$ are not intrinsically satisfied by the solutions (similarly to what happens in direct tomography of a density matrix).

A different approach can be devised by following a Maximum Likelihood (ML) estimation method. The main idea is to parametrize the correlation matrix in such a way that the physical constraints are intrinsically satisfied. One observes that the $C_{i,j}$ for a generic two-fermion state, written as $\rho = \sum_\alpha p_\alpha |\psi_\alpha\rangle\langle\psi_\alpha|$ satisfies the following properties:

(p.1) $\text{Tr}(C) = 2$. This property is obtained due to the constraint $\sum_i N_i = 2$.

(p.2) $C_{i,j} = C_{j,i}^*$ and $\text{Im}(C_{i,i}) = 0$. Hence, the correlation matrix is Hermitian, and can be thus diagonalized obtaining real eigenvalues.

(p.3) Since $\langle d_i^\dagger d_i \rangle \geq 0$ for all set of orthogonal modes $\{d_i\}$, all the eigenvalues λ_i of the correlation matrix are $\lambda_i \geq 0$, and thus $C_{i,j}$ is non-negative.

(p.4) For fermionic states the eigenvalues λ_i are bounded to be $\lambda_i \leq 1$ due to the Pauli exclusion principle, since the eigenvalues represent the average number of particles $\langle \tilde{d}_i^\dagger \tilde{d}_i \rangle$ in the eigenmodes $\tilde{d}_i$.

Starting from property (p.2), the correlation matrix can be parametrized by using the spectral decomposition. A complex-valued Hermitian matrix can be decomposed as $C = EDE^*$, where D is a real-valued diagonal matrix whose elements represent the eigenvalues of C, and E is a unitary matrix whose columns are the eigenvectors of C. The constraint imposed by properties (p.1), (p.3) and (p.4) can be directly imposed on the eigenvalues $\{\lambda_i\}$. Property (p.1) can be directly ensured by expressing the eigenvalues as a function of a set of non-negative real-valued parameters $\{t_i\}$: $\lambda_i = 2t_i / \sum_k t_k$. Properties (p.3) and (p.4) can be included by performing a constrained minimization of the likelihood function. In parallel, the unitary matrix E can be parametrized as a function of a set of real-valued parameters $\{\omega_i, \phi_i\}$ by exploiting Reck's lemma

[13], or equivalently by applying the recipe of Ref. [14]. According to this approach, a general $m \times m$ unitary matrix can be decomposed as a product of 2×2 transformations by using $m(m-1)$ real parameters. The complete set of parameters $\{t_i, \omega_i, \phi_i\}$ can be in principle retrieved by a Maximum Likelihood approach, thus obtaining the values $\{\tilde{t}_i, \tilde{\omega}_i, \tilde{\phi}_i\}$ that minimizes the distance between the experimental data and the prediction from the theoretical model. In the present case, the likelihood function can be defined as:

$$\mathcal{L} = \sum_{i=1}^{5} \frac{(|C_{i,i}| - \tilde{N}_{i,i})^2}{(\delta \tilde{N}_{i,i})^2} + \sum_{i=2}^{5}\sum_{j=1}^{i-1} \frac{(|C_{i,j}| - \tilde{C}^{(\mathrm{m})}_{i,j})^2}{(\delta \tilde{C}^{(\mathrm{m})}_{i,j})^2} + \frac{(\Re(C_{1,5}) - \frac{\tilde{N}'_5 - \tilde{N}'_1}{2})^2}{\frac{1}{4}(\delta \tilde{N}'_5)^2 + \frac{1}{4}(\delta \tilde{N}'_1)^2} + \frac{(\Re(C_{2,4}) - \frac{\tilde{N}'_2 - \tilde{N}'_4}{2})^2}{\frac{1}{4}(\delta \tilde{N}'_2)^2 + \frac{1}{4}(\delta \tilde{N}'_4)^2}, \tag{73}$$

where $\tilde{N}_i$ is obtained according to Eq. (72), $\tilde{C}^{(\mathrm{m})}_{i,j} = (\tilde{N}_i \tilde{N}_i - 2\tilde{P}_{i,j})^{1/2}$ with corresponding error $\delta \tilde{C}^{(\mathrm{m})}_{i,j}$, and $\tilde{N}'_i$ is obtained according to Eq. (72) from the measured distribution after the ECC chip $\tilde{P}'_{i,j}$.

We observe that the measured data do not provide a complete set, since no information is retrieved on some of the complex phases. Hence, we expect that the likelihood function $\mathcal{L}$ will present several equivalent minima. These minima will correspond to a set $\mathcal{M}_1$ of different correlation matrices, having the same moduli and different complex phases. Numerically, this means that the minimization of the likelihood function $\mathcal{L}$ will give several equivalent minima depending on the initial condition of the adopted local optimization routine. This dependence on the starting point can be exploited to determine numerically an upper and a lower bound on the purity and on the entanglement quantifiers of interest. We then evaluated according to this approach $N_1 = 10^3$ equivalent correlation matrices, where the value of the likelihood functions differ by $\Delta\mathcal{L}/\mathcal{L} < 10^{-3}$ from the obtained global minimum $\mathcal{L}_{\min} \sim 10.606$. In parallel, the effect of experimental errors on the estimated correlation matrix can be obtained by a MonteCarlo approach. We numerically generated $N_2 = 10^3$ datasets, normally distributed with mean and variance equal to the experimental values, and then evaluated a set $\mathcal{M}_2$ of N_2 different correlation matrices.

Starting from these results, we then evaluated the purity Ψ of the QTC's output. The calculation of the purity of the system is performed starting from the assumption that the output state is a Gaussian (fermionic) state. In that case $\rho = \frac{1}{Z}\exp\left(-\sum h_{ij} c_i^\dagger c_j\right)$. To simplify the calculation we perform a bogoliubov transformation to write the above expression in terms of independent modes $\rho = \frac{1}{Z}\exp\left(-\sum \omega_k d_k^\dagger d_k\right)$ where the d_k are the new annihilation operators. It is simple to show that $Z = \prod_k (1 + e^{\omega_k})$ and that $e^{\omega_k} = \frac{\zeta_k}{1-\zeta_k}$ where ζ_k are the eigenvalues of the $N \times N$ matrix $\tilde{C}_{ij} = \langle c_i^\dagger c_j \rangle$. Therefore the purity Ψ is

$$\Psi = \mathrm{Tr}[\rho^2] = \prod_k \left[(1-\zeta_k)^2 + \zeta_k^2\right] = \det\left[(\mathbb{1} - \tilde{C})^2 + \tilde{C}^2\right] \ . \tag{74}$$

where $(A^2)_{ij} = \sum_k A_{ik}A_{kj}$ is the matrix power. The interval on the purity Ψ obtained from the set $\mathcal{M}_2$ is found to be two orders of magnitude greater than the interval obtained from the set $\mathcal{M}_1$ of correlation matrices (see Supplementary Fig. 13a). Thus, the main contribution on the purity is due to experimental errors, and we will neglect the set $\mathcal{M}_1$. In conclusion, the value of the purity is found to be $\tilde{\Psi} = 0.943 \pm 0.017$.

In parallel, the amount of entanglement present in the QTC's output can be quantified by exploiting the "hashing inequality" [15]. The latter provides a lower bound on the amount of distillable entanglement from a given bipartition of the system according to the inequality:

$$\mathrm{ED}_{\mathrm{A,B}} > S(\rho_{\mathrm{A}}) - S(\rho_{\mathrm{AB}}) = \overline{\mathrm{ED}}_{\mathrm{A,B}}, \tag{75}$$

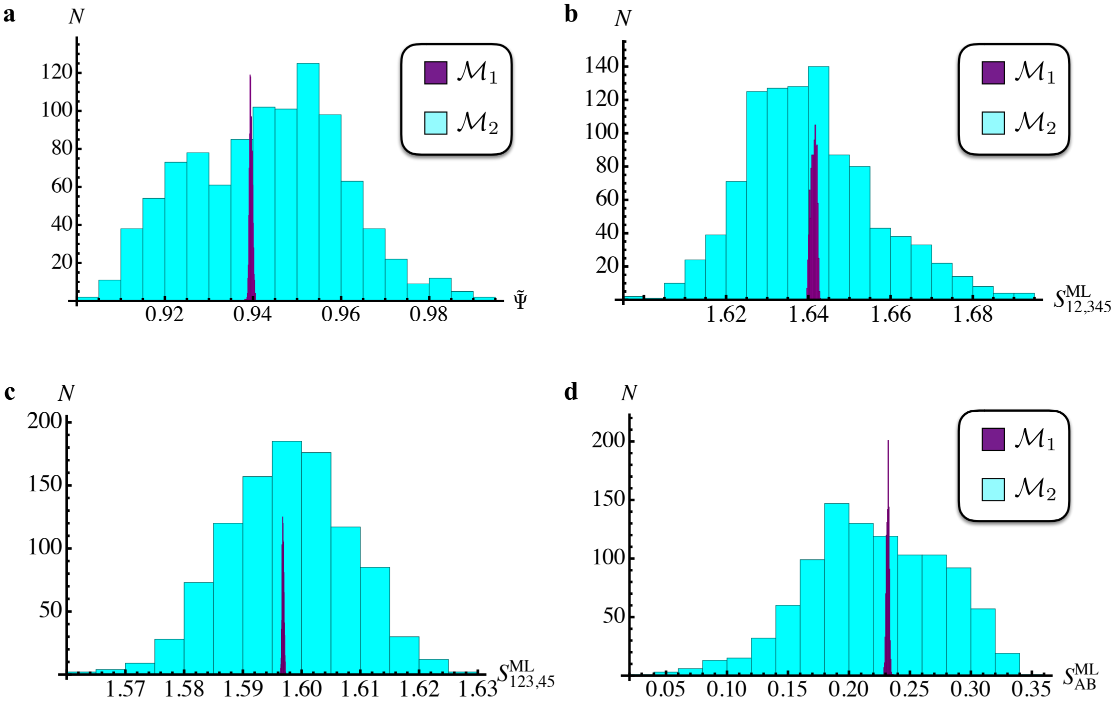

Supplementary Figure 13. Histogram of the purities $\tilde{\Psi}$ (**a**), of the entanglement entropy $S^{\mathrm{ML}}_{12,345}$ (**b**), of the entanglement entropy $S^{\mathrm{ML}}_{123,45}$ (**c**), and of the overall entropy S^{ML} (**d**) for the correlation matrices belonging to sets $\mathcal{M}_1$ and $\mathcal{M}_2$.

where AB is the overall system, A is one of the two subsystem of a given bipartition, and S stands for the von Neumann entropy. The entanglement entropy $S(\rho_{\mathrm{A}})$ can be directly evaluated as discussed in the previous section, or alternatively can be retrieved from the eigenvalues of the reduced correlation matrices obtained via the ML procedure discussed above. Finally, the overall entropy $S_{\mathrm{AB}} = S(\rho_{\mathrm{AB}})$ can be obtained according to Eq. (64) from the full correlation matrix. By considering only the set $\mathcal{M}_2$ (see Supplementary Fig. 13b-d), the entropies estimated from the ML correlation matrices read $S^{\mathrm{ML}_{\mathrm{AB}}} = 0.22 \pm 0.05$ for the overall state, $S^{\mathrm{ML}}_{12,345} = 1.640 \pm 0.016$ and $S^{\mathrm{ML}}_{123,45} = 1.597 \pm 0.010$ for the subsystems. Finally, the lower bounds $\overline{\mathrm{ED}}_{12,345}$ and $\overline{\mathrm{ED}}_{123,45}$ for two different bipartions are found to be $\overline{\mathrm{ED}}_{12,345} = 1.42 \pm 0.06$ and $\overline{\mathrm{ED}}_{123,45} = 1.38 \pm 0.06$ respectively.

Convergence of the Maximum Likelihood approach

Here we briefly discuss the convergence of the ML procedure discussed in the previous section. More specifically, we discuss the role of the parameter $\Delta\mathcal{L}/\mathcal{L}$ in determining the set $\mathcal{M}_1$ of correlation matrices. In the discussion above, we considered to be equivalent the correlation matrices which present a relative difference in the likelihood function $\Delta\mathcal{L}/\mathcal{L} < 10^{-3}$. This choice has to be motivated with respect to the set $\mathcal{M}_2$ obtained by performing a MonteCarlo simulation on the

experimental errors.

In principle, the ML procedure should determine all correlation matrices presenting the same value of the likelihood function $\mathcal{L}$. This procedure is performed by changing the initial point in a local minimization routine to explore the local minima of $\mathcal{L}$. Due to the significant number of involved parameters, this approach reveals to be computationally expensive, since not all local minima present the same value of $\mathcal{L}$. Thus, we can relax the condition by accepting all local minima presenting a relative difference $\Delta\mathcal{L}/\mathcal{L}$ lower than a threshold ϵ with respect to the global minimum. To motivate the performed choice $\epsilon = 10^{-3}$, we have repeated the ML approach by considering different values for the threshold ϵ imposed on the likelihood function. The results for the complex phases of the off-diagonal elements of $C_{i,j}$ are shown Supplementary Figs. 14-15.

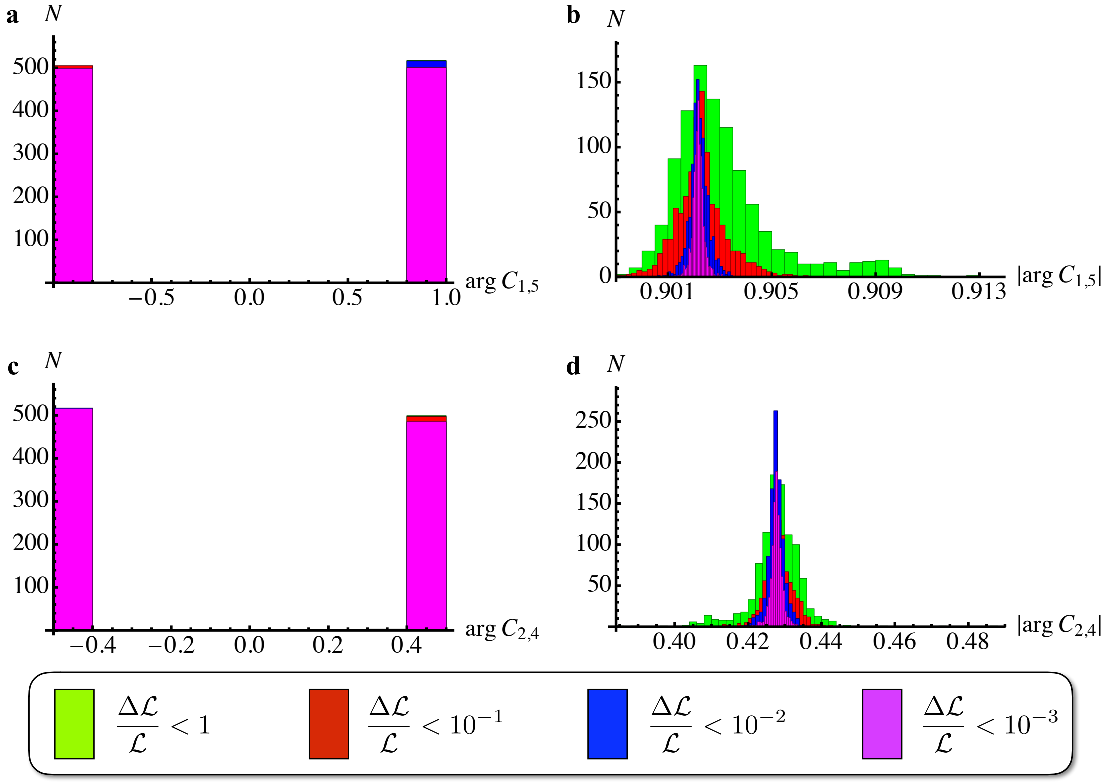

Supplementary Figure 14. Histograms of $\arg C_{1,5}$ (**a**), $|\arg C_{1,5}|$ (**b**), $\arg C_{2,4}$ (**c**), $|\arg C_{2,4}|$ (**d**) for different values of the threshold imposed on $\Delta\mathcal{L}/\mathcal{L}$ in the ML estimation procedure (see legend).

Concerning the elements $C_{1,5}$ and $C_{2,4}$, partial information is available from the experimental measurements as discussed above. In this case, a lower value of the threshold ϵ corresponds to a reduced interval for $|\arg C_{i,j}|$ (see Supplementary Fig. 14), since less tolerance is imposed by the algorithm with respect to the experimental estimates (similar results are obtained for the moduli $|C_{i,j}|$). Note that the presence of two equivalent peaks with opposite signs in histograms of Supplementary Figs. 14a,c is due to the lack of information on the sign of $\arg C_{i,j}$.

Conversely, for all other off-diagonal elements $C_{i,j}$, with $(i, j) \neq (1, 5) \neq (2, 4)$, a different be-

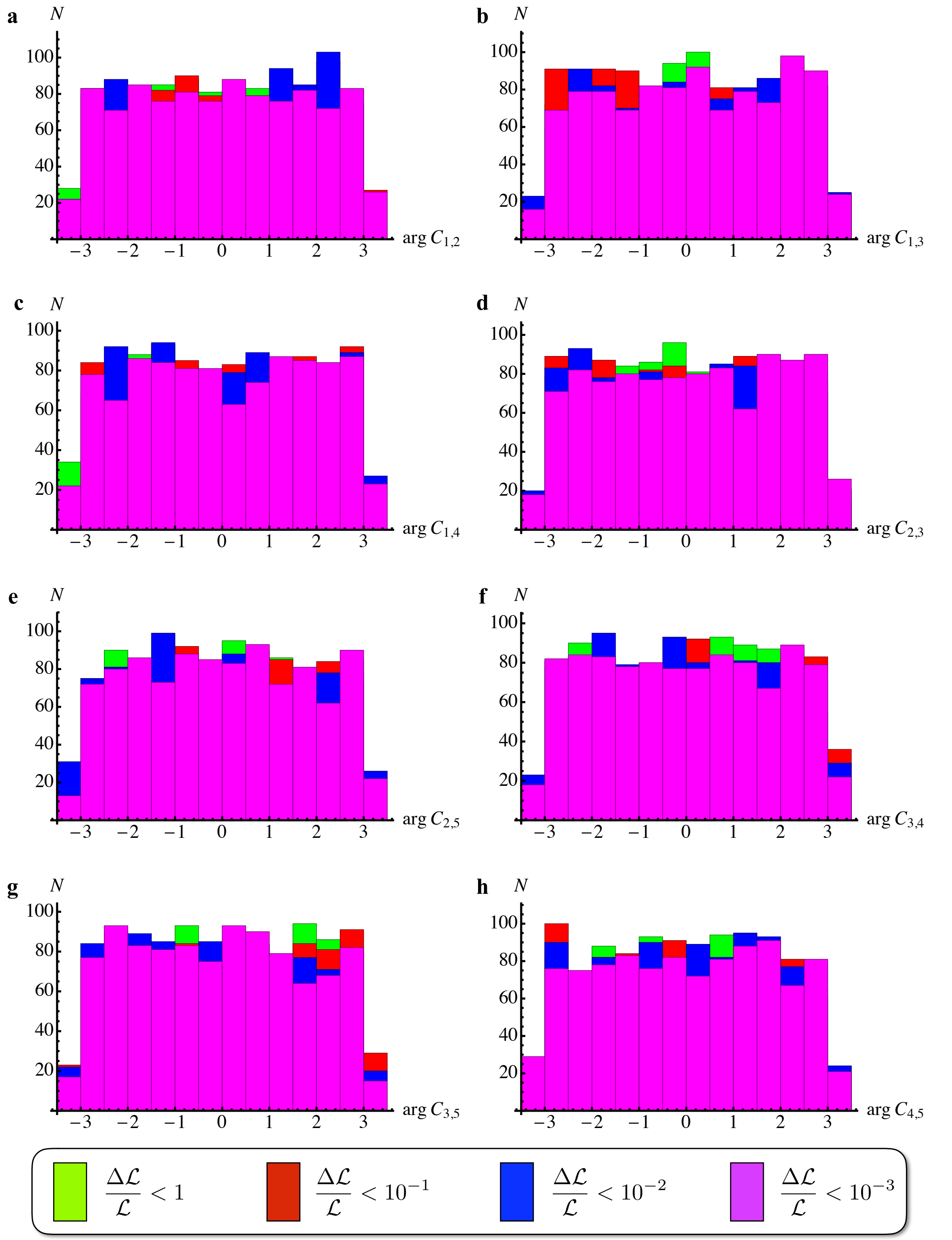

Supplementary Figure 15. Histograms of $\arg C_{1,2}$ (**a**), $\arg C_{1,3}$ (**b**), $\arg C_{1,4}$ (**c**), $\arg C_{2,3}$ (**d**), $\arg C_{2,5}$ (**e**), $\arg C_{3,4}$ (**f**), $\arg C_{3,5}$ (**g**), $\arg C_{4,5}$ (**h**), for different values of the threshold imposed on $\Delta\mathcal{L}/\mathcal{L}$ in the ML estimation procedure (see legend).

haviour is obtained (see Supplementary Fig. 15). Indeed, an almost flat distribution in the whole interval $[-\pi, \pi]$ is observed. This results depends on the lack of information on these elements. Hence, the ML procedure discussed above spans the complete interval $\arg C_{i,j}$ independently from the threshold ϵ.

Since the main contribution in the uncertainties on the entanglement quantifiers arises from the experimental errors, it is sufficient to consider a threshold equal to $\epsilon = 10^{-3}$.

SUPPLEMENTARY NOTE 12: TOWARDS MEASURING THE ENTANGLEMENT ENTROPY IN INTERACTING (NON-LINEAR) SYSTEMS

In the previous section we have shown how to measure the von Neumann entanglement entropy by exploiting the Wick's theorem, namely the Gaussian nature of the resulting state. However, in the presence of non-linearities the evolved state becomes non-Gaussian, so the previous analysis cannot be applied. Here we show how our approach can be generalized in certain cases also to non-linear models, by extending the methods of Refs. [16,17]. Following those schemes we use the Renyi entropy $S^2_{\mathrm{A:B}}$ as a measure of entanglement between two blocks A and B

$$S^2_{\mathrm{A:B}} = -\log_2 \mathrm{Tr}[\rho_{\mathrm{A}}^2] = -\log_2 \mathrm{Tr}[\rho_{\mathrm{B}}^2]. \tag{76}$$

The latter quantity can be measured using two copies of the same physical system $\rho_{\mathrm{AB}} \otimes \rho_{\mathrm{AB}}$ by measuring the SWAP operator

$$V_{\mathrm{A}} = \prod_{i \in \mathrm{A}} \mathrm{SWAP}_i, \tag{77}$$

$$\mathrm{SWAP}_i = \frac{1}{2}\left(\mathbb{1} \otimes \mathbb{1} + \sum_{\alpha = X,Y,Z} \sigma_i^\alpha \otimes \sigma_i^\alpha\right). \tag{78}$$

Indeed, it is simple to show that

$$S^2_{\mathrm{A:B}} = -\log_2 \langle V_{\mathrm{A}} \rangle, \tag{79}$$

where the expectation value is taken with respect to the state $\rho_{\mathrm{AB}} \otimes \rho_{\mathrm{AB}}$.

We now show how to measure V_{A} in two copies of a spin chain of L simulated spins. For convenience we label the spins of the first copy in decreasing order, while the spins of the second copy are in increasing order. We consider a continuous bipartition $\mathrm{A} \cup \mathrm{B}$ of the chain and we call A_i, B_i $(i = 1, 2)$ the bipartition in each copy, so that the two copies are organized as $\mathrm{B}_1\mathrm{A}_1\mathrm{A}_2\mathrm{B}_2$ where the order of the spins in A_1 and B_1 is reversed. Therefore, the first spin of A_1 and the first spin of A_2 are nearest neighbours (in position L and $L+1$). Because of this, thanks to the Jordan-Wigner transformation

$$\mathrm{SWAP}_1 = \frac{1}{2}\left(\mathbb{1} + \sum_{\alpha = X,Y,Z} \sigma_L^\alpha \sigma_{L+1}^\alpha\right) \tag{80}$$

$$= 2c_L^\dagger c_{L+1}^\dagger c_{L+1} c_L + c_{L+1}^\dagger c_L + c_L^\dagger c_{L+1} - c_{L+1}^\dagger c_{L+1} - c_L^\dagger c_L + \mathbb{1} \tag{81}$$

$$= \mathrm{BS}_{L,L+1}^\dagger (2c_L^\dagger c_{L+1}^\dagger c_{L+1} c_L - 2c_{L+1}^\dagger c_{L+1} + \mathbb{1}) \mathrm{BS}_{L,L+1}. \tag{82}$$

Because of the above identity, when A is composed of a single spin (in the first position), the measurement of $V_{\rm A}$ can be done with the same technology discussed in this paper. Indeed, after having prepared two copies of the system, one has to use an ECC chip to perform the BS operation between modes L and $L+1$ and then measure the expectation values (41). Indeed,

$$\langle \mathrm{SWAP}_1 \rangle = 2P'_{L,L+1} - 2N'_{L+1} + 1 \tag{83}$$

where the prime refers to operators after the ECC chip, namely after the beam splitter transformation.

For other pairs of spins one has to consider the effect of the Jordan-Wigner string. For instance

$$\mathrm{SWAP}_2 = \frac{1}{2}\left(\mathbb{1} + \sum_{\alpha=X,Y,Z} \sigma^{\alpha}_{L-1} \otimes \sigma^{\alpha}_{L+2}\right) \tag{84}$$

$$= \mathrm{BS}^{\dagger}_{L-1,L+2}(2c^{\dagger}_{L-1}c^{\dagger}_{L+2}c_{L+2}c_{L-1} - 2c^{\dagger}_{L+2}c_{L+2} + \mathbb{1})\mathrm{BS}_{L-1,L+2}\Phi_1, \tag{85}$$

where

$$\Phi_i = \sigma^{Z}_{L-i+1}\sigma^{Z}_{L+i} = 4c^{\dagger}_{L-i+1}c^{\dagger}_{L+i}c_{L+i}c_{L-i+1} - 2c^{\dagger}_{L+i}c_{L+i} - 2c^{\dagger}_{L-i+1}c_{L-i+1} + \mathbb{1}. \tag{86}$$

One can see that Φ_i is invariant with respect to the BS transformation, i.e. $\mathrm{BS}^{\dagger}_{L-i+1,L+i}\Phi_i\mathrm{BS}_{L-i+1,L+i} = \Phi_i$, and that

$$\mathrm{SWAP}_1\Phi_1 = \mathrm{BS}^{\dagger}_{L,L+1}(2c^{\dagger}_{L}c^{\dagger}_{L+1}c_{L+1}c_{L} - 2c^{\dagger}_{L}c_{L} + \mathbb{1})\mathrm{BS}_{L,L+1}. \tag{87}$$

Generalizing the above argument one finds that

$$V_{\rm A} = \prod_{i\in \rm A} \mathrm{BS}^{\dagger}_{L-i+1,L+i}\, V_i\, \mathrm{BS}_{L-i+1,L+i} \tag{88}$$

where each V_i can be expressed in terms of diagonal operators in the Fock basis. Their explicit form is

$$V_i = 2c^{\dagger}_{n}c^{\dagger}_{m}c_{m}c_{n} - 2c^{\dagger}_{m}c_{m} + \mathbb{1}, \tag{89}$$

for either $n = L-i+1, m = L+i$ or $m = L-i+1, n = L+i$, depending on the parity of i. The final expectation value $\langle V_{\rm A}\rangle$ is then obtained with a two-step procedure similar to the one performed in the experiment presented in this paper: (i) one has to use an ECC chip to perform the beam splitter operations entering in (88); (ii) from the output modes of the ECC chip one has to use photodetectors to measure some operators

$$P_{n_1,n_2,\ldots,n_a} = \langle c^{\dagger}_{n_1}c^{\dagger}_{n_2}\ldots c^{\dagger}_{n_a}c_{n_a}\ldots c_{n_2}c_{n_1}\rangle\ , \tag{90}$$

namely those entering in the product $\prod_i V_i$. The different $P_{n_1,n_2,\ldots,n_a}$ can be obtained by measuring a-fold coincidences at the output of the ECC chip. Such detection procedure is performed conditioned to measuring a number of output photons equal to the input ones. This allows to overcome the effect of optical losses in the system, which will result only in a reduction of the detected signal.

SUPPLEMENTARY REFERENCES

[1] Banchi, L. Ballistic quantum state transfer in spin chains: General theory for quasi-free models and arbitrary initial states. *Eur. Phys. J. Plus* **128**, 137 (2013).
[2] Strauch, F. W. Connecting the discrete- and continuous-time quantum walks. *Phys. Rev. A* **74**, 030301 (2006).
[3] Kurzyński, P. & Wójcik A. Discrete-time quantum walk approach to state transfer. *Phys. Rev. A* **83**, 062315 (2011).
[4] Christandl, M., Datta, N., Ekert, A. & Landahl A. J. Perfect state transfer in quantum spin networks. *Phys. Rev. Lett.* **92**, 187902 (2004).
[5] Banchi, L., Apollaro, T., Cuccoli, A., Vaia, R. & Verrucchi, P. Long quantum channels for high-quality entanglement transfer. *New J. Phys.* **13**, 123006 (2011).
[6] Alkurtass, B., Banchi, L. & Bose, S. Optimal quench for distance-indipendent entanglement and maximal block entropy. *Phys. Rev. A* **60**, 042304 (2014).
[7] Rafiee, M., Lupo, C. & Mancini, S., Noise to lubricate qubit transfer in a spin network. *Phys. Rev. A* **88**, 032325 (2013).
[8] Wilcox, R. M. Exponential operators and parameter differentiation in quantum physics. *J. Math. Phys.* **8**, 962-982 (1967).
[9] Hong, C. K., Ou, Z. Y. & Mandel, L. Measurement of subpicosecond time intervals between two photon by interference. *Phys. Rev. Lett.* **59**, 2044-2046 (1987).
[10] Laing, A. & O'Brien, J. L. Super-stable tomography of any linear optical device. Preprint at http://arxiv.org/abs/1208.2868v1 (2012).
[11] Crespi, A., Osellame, R., Ramponi, R., Bentivegna, M., Flamini, F., Spagnolo, N., Viggianiello, N., Innocenti, L., Mataloni, P. & Sciarrino, F. Suppression law of quantum states in a 3D photonic fast Fourier transform chip. *Nature Commun.* **7**, 10469 (2016).
[12] Peschel, I. & Eisler, V. Reduced density matrices and entanglement entropy in free lattice mode. *J. Phys. A: Math. Theor.* **42**, 504003 (2009).
[13] Reck, M., Zeilinger, A., Bernstein, H. J. & Bertani, P. Experimental realization of any discrete unitary operator. *Phys. Rev. Lett.* **73**, 58-61 (1994).
[14] Clements, W. R., Humphreys, P. C., Metcalf, B. J., Kolthammer, W. S., & Walmsley, I. A. Optimal design for universal multiport interferometers. *Optica* **3**, 1460-1465 (2016).
[15] Horodecki, R., Horodecki, P., Horodecki, M., & Horodecki, K. Quantum entanglement. *Rev. Mod. Phys.* **81**, 865-942 (2009).
[16] Moura Alves, C. & Jaksch, D. Multipartite entanglement detection in bosons. *Phys. Rev. Lett.* **93**, 110501 (2004).
[17] Pichler, H., Bonnes, L., Daley, A. J., Läuchli, A. M. & Zoller, P. Thermal versus entanglement entropy: a measurement protocol for fermionic atoms with a quantum gas microscope. *New J. Phys.* **15**, 063003 (2013).
